# Triple-Band Warm White-Light Emission from Type II Band-Aligned Aggregation-Induced Enhanced Emission Organic Cation-Incorporated Two-Dimensional Lead Iodide Perovskite

**DOI:** 10.3390/ijms26115054

**Published:** 2025-05-24

**Authors:** Almaz R. Beisenbayev, Igor Ivanov-Prianichnikov, Anatoly Peshkov, Tangsulu Adil, Davit Hayrapetyan, Chang-Keun Lim

**Affiliations:** 1 Department of Chemical and Materials Engineering, School of Engineering and Digital Sciences, Nazarbayev University, Astana 010000, Kazakhstan; almaz.beisenbayev@nu.edu.kz (A.R.B.); anatolii.peshkov@nu.edu.kz (A.P.); tangsulu.adil@nu.edu.kz (T.A.); 2 Department of Chemistry, School of Sciences and Humanities, Nazarbayev University, Astana 010000, Kazakhstan; igor.ivanovpryanichnikov@nu.edu.kz

**Keywords:** aggregation-induced emission (AIE), 2D perovskite, photoluminescence, white luminescence, self-trapped exciton, energy transfer

## Abstract

Single-phase white-light-emitting materials, particularly 2D hybrid organic–inorganic halide perovskites, have garnered significant attention due to their strong electron–phonon interactions, which lead to broad luminescence and a notable Stokes shift resulting from self-trapped exciton recombination. However, 2D lead iodide perovskites typically display these characteristics poorly, restricting their efficiency as white-light emitters. This study presents a 2D lead iodide perovskite that incorporates a fluorinated π-conjugated aggregation-induced enhanced emission luminophore, FPCSA, as a bulky organic cation to create a quasi-2D perovskite. The FPCSA cation establishes a Type II energy level alignment with the lead iodide layer in the 2D perovskite, and a significant energy offset effectively suppresses charge transfer, enabling independent emission from both the organic and inorganic layers while facilitating self-trapped exciton formation. Under 315 nm UV excitation, this material demonstrates warm white-light emission with RGB triple-band photoluminescence stemming from the electronically decoupled FPCSA and perovskite layers. These findings provide a promising new method for designing efficient single-phase white-light-emitting materials for optoelectronic applications.

## 1. Introduction

The development of white-light-emitting diodes (WLEDs) has become a significant area of research due to their wide applications in full-color flat-panel displays and solid-state lighting, which contribute to both energy savings and device miniaturization [1,2,3,4]. Traditionally, WLEDs are fabricated by combining a blue LED with a yellow phosphor or by integrating separate red, green, and blue (RGB) LEDs [5,6]. However, systems that use multiple emitters often experience color instability over time due to the differing degradation rates of each component, along with decreased efficiency caused by reabsorption, which arises from spectral overlap between the absorption and emission bands of the individual emitters [6,7,8,9]. A promising alternative to these multi-component systems is the development of single-phase white-light-emitting materials, which eliminate the need for complex integration processes and reduce issues related to reabsorption losses by ensuring a continuous broadband emission spectrum that spans the entire visible range (400–700 nm) [10,11,12]. Various classes of materials, such as doped oxides [13,14,15], organic compounds [16,17,18], metal–organic frameworks [19,20,21], and perovskites [22,23,24], have been explored for this purpose. Among them, lead- and tin-based halide perovskites stand out due to their exceptional photoluminescence quantum efficiencies and tunable color emission, making them promising candidates for high-performance LEDs [25,26,27]. In parallel, two-dimensional perovskites (2D PVSKs) have emerged as versatile platforms for solution-processable optoelectronics, offering enhanced structural flexibility compared to conventional 3D perovskites [28,29]. Some 2D perovskites exhibit pronounced polaron localization effects, resulting in broad emission with an unusually large Stokes shift, which helps reduce luminescence reabsorption [29,30].

Expanding on this potential, efficient white-light emission from two-dimensional (2D) layered perovskites, such as (N-MEDA)PbX_4_ and (EDBE)PbX_4_ (where X = Cl or Br), has been demonstrated, highlighting their capability as single-phase white-light emitters [22]. Two-dimensional perovskites feature a flexible lattice structure and exhibit strong electron–phonon coupling, which often induces local lattice distortions and facilitates the formation of self-trapped states within the bandgap [22,29,30,31]. In 2D PVSKs, charge carriers are typically confined within lead halide layers, resulting in high exciton binding energy, a large bandgap, and narrow emission bands from free excitons (FEs). These free excitons eventually relax into self-trapped states, giving rise to self-trapped excitons (STEs). Unlike FEs, STEs emit at lower energies and are characterized by broad photoluminescence spectra, with a full width at half maximum (FWHM) exceeding 100 nm and a significant Stokes shift of several hundred meV, due to their localized nature. The emission intensity of STEs is primarily governed by the potential barrier height and the transformation dynamics between FEs and STEs. The material’s dimensionality and electron–phonon coupling strength dictate the barrier height, while temperature regulates the transition between these states [32]. As a result, STE formation and emission properties in 2D and quasi-2D PVSKs are strongly dependent on the number of inorganic perovskite layers (n-value). The n-value determines the degree of quantum and dielectric confinement, directly influencing exciton behavior, electron–phonon interactions, and lattice distortions [33]. In single-layer quasi-2D PVSKs (n = 1), excitons experience strong quantum confinement within single [PbX_6_]^4−^ layers, leading to large exciton binding energies (>200 meV) and enhanced electron–phonon coupling. The highly flexible lattice structure allows for significant distortions, promoting STE formation and resulting in dominant, red-shifted emission with a broad spectral profile and large Stokes shifts. As the layer number increases in quasi-2D PVSKs (n = 2–5), the degree of quantum confinement decreases, leading to reduced exciton binding energy and increased structural rigidity. This diminishes lattice distortions and lowers the probability of STE formation. Consequently, a competitive interplay emerges between FE and STE emissions, where lower-n perovskites exhibit STE-dominated emission, while higher-n perovskites show a mixture of FE and STE emissions or primarily FE emission. At even higher n-values (n ≥ 5), the system approaches bulk-like behavior, significantly weakening electron–phonon coupling and favoring free exciton recombination over STE formation. This results in a blue-shifted emission spectrum and a substantial reduction in the contribution of STE emission [29,30,31,32,33].

For perovskites, white-light emission is typically achieved using L_2_PbBr_x_Cl_4−x_ formulations (where L represents a large organic cation). The halide composition significantly influences the emission profile. L_2_PbCl_4_ mainly shows a single broad emission band due to strong electron–phonon coupling and deep self-trapped states, which leads to dominant STE [23,34]. In contrast, L_2_PbBr_4_, with weaker electron–phonon coupling and less lattice distortion, primarily exhibits FE emission (~400–450 nm), without significant STE contribution [34,35]. When Br^−^ and Cl^−^ are mixed in L_2_PbBr_x_Cl_4−x_, intermediate lattice distortions allow for both FE and STE emissions, producing a broad, warm white-light spectrum [22,35]. Iodine-based analogs have inherently narrower bandgaps and less lattice distortion, leading to pronounced FE and weak STE emissions, typically observed in green and red spectral regions at room temperature [36]. However, STE emission can be enhanced by tailoring exciton–phonon interactions through precise ligand engineering [37,38].

To achieve white luminescence from iodine-based quasi-2D perovskites (PVSKs), we consider incorporating rigid π-conjugated luminophores (LPs) into the perovskite lattice to introduce blue luminescence and lattice distortion. In 2D PVSKs, long-chain aliphatic and small aromatic organic cations are commonly used as insulating layers to spatially confine photo-generated excitons within the quantum wells of 2D perovskites [28,39]. Recently, π-conjugated luminophore-based organic cations have been introduced to simultaneously improve the optical and electronic properties of these materials [40,41,42,43]. However, due to the inherent photoluminescence quenching associated with Dexter and/or Förster energy transfer between adjacent luminophores, π-conjugated organic cations that exhibit aggregation-induced enhanced emission (AIEE) behavior have been proposed and demonstrated as a viable strategy for overcoming these challenges.

AIEE LPs represent a new class of LPs, demonstrating advanced PL properties with an increased concentration of LPs [44,45]. Unlike conventional LPs, AIEE LPs inherently resist concentration-induced quenching [46], facilitating prolonged exciton lifetimes within the AIEE organic cation layer. This enhancement, in turn, increases the likelihood of efficient energy or charge transfer between the organic and 2D PVSK layers. The presence of AIE cations can introduce additional distortions in the [PbI_6_]^4−^ octahedral framework, which strengthens exciton–phonon interactions and can also affect dielectric screening in 2D perovskites, altering the exciton binding energy and favoring self-trapping mechanisms. In our previous work [47], we demonstrated a significant improvement in both linear and nonlinear optical properties of 2D AIEE-PVSK materials ((AIEE)_2_MA_n−1_Pb_n_I_3n+1_ (n = 2, 3, 4), where AIEE denotes the AIEE cation and MA represents methylammonium), driven by interlayer sensitization from the AIEE layer. The designed AIEE cation ((Z)-2-([1,1′-biphenyl]-4-yl)-3-(4-(3-aminopropoxy)phenyl)acrylonitrile, BPCSA) seamlessly integrated into the perovskite lattice, forming stable quasi-2D perovskite structures with superior optical characteristics compared to conventional quasi-2D perovskites incorporating long-chain alkylammonium cations. BPCSA_2_MA_n−1_Pb_n_I_3n+1_ exhibited Type I energy level alignment between BPCSA and the 2D PVSK layers, where the organic layer formed a Frenkel-type exciton band that could demonstrate Forster/Dexter energy transfer to the inorganic layer, while the inorganic layer showed the formation of Wannier excitons. However, single-layered AIEE_2_PbI_4_ was neither synthesized nor characterized, and a STE band was not observed. Moreover, the Type I band alignment eliminated the blue emission from the AIEE LPs through energy transfer, so we need a strategy to prevent this energy transfer to achieve white luminescence with AIEE-2D PVSK.

**Figure 1:**
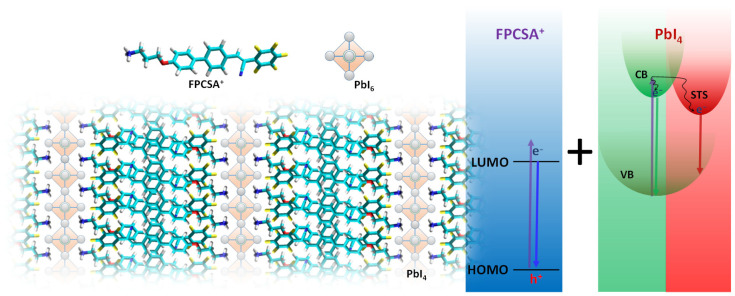
Schematic representation of the AIEE luminophore (FPCSA), its assembled FPCSA_2_PbI_4_ 2D PVSK, and the photophysical process of isolated RGB triple-band emission from the quasi-2D PVSK.

In this study, we present an approach where a single-layered 2D perovskite incorporates a fluorinated π-conjugated AIEE ligand, FPCSA, which features a highly electronegative fluorophenyl fragment that alters HOMO-LUMO levels. The synthesized FPCSA exhibits structural compatibility with the perovskite lattice and forms a deep type II band alignment with the PbI_4_ phase, effectively suppressing charge transfer. This enables independent emissions from both the AIEE dye and the 2D perovskite phase while enhancing STE generation in the perovskite. As a result, a triple-band emission is achieved, covering blue, green, and red wavelengths, originating from AIEE LP, 2D perovskite FE, and STE, respectively, ultimately producing warm white-light emission under 315 nm excitation at room temperature (Figure 1).

## 2. Results and Discussion

**Figure 2:**
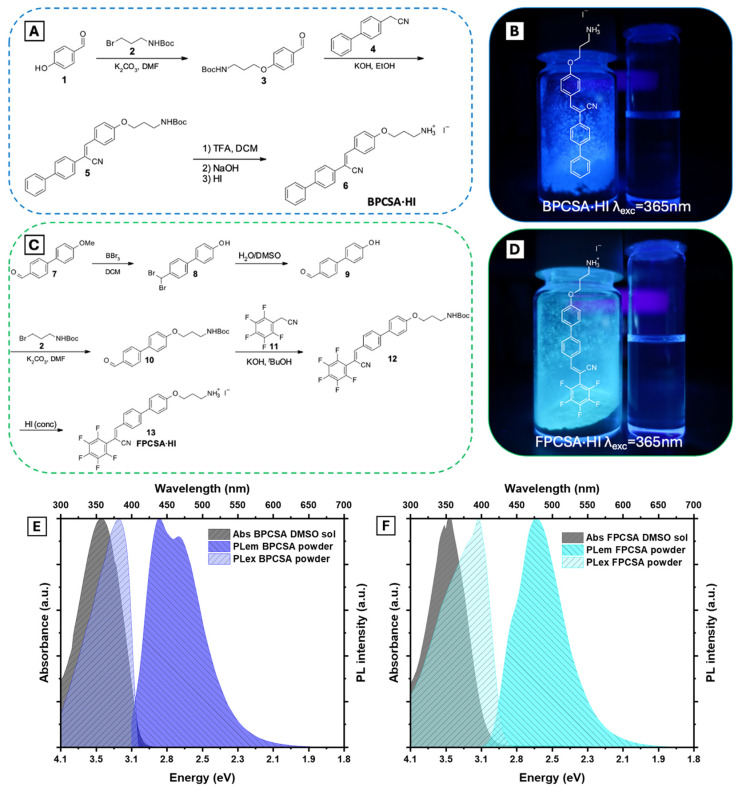
(**A**)—Synthetic pathway and (**B**)—photograph of BPCSA·HI powder and solution under 365 nm excitation. (**C**)—Synthetic pathway and (**D**)—photograph of FPCSA·HI powder and solution under 365 nm excitation. (**E**,**F**)—PL emission and excitation spectra of BPCSA·HI and FPCSA·HI powders, respectively, along with UV-vis absorption spectra of the corresponding LPs in dimethyl sulfoxide (DMSO).

To demonstrate white luminescence in lead–iodide-based single layers of AIEE quasi-2D PVSK, we selected two AIEE LP cations. First, we chose (Z)-2-([1,1′-biphenyl]-4-yl)-3-(4-(3-aminopropoxy)phenyl)acrylonitrile (BPCSA, Figure 2A) from our previous study to evaluate its potential STE band in single-layer quasi-2D PVSK. Additionally, a fluorinated AIEE LP, (Z)-3-(4′-(3-aminopropoxy)-[1,1′-biphenyl]-4-yl)-2-(perfluorophenyl)acrylonitrile (FPCSA, Figure 2C), was designed to potentially form a Type II (or III) band alignment at the LP and lead halide single-layer interface. Compared to non-fluorinated LPs, fluorine substitution in π-conjugated LPs lowers both HOMO and LUMO energy levels due to its high electronegativity [48,49]. BPCSA·HI and FPCSA·HI were synthesized following the routes depicted in Figure 2A,C. The BPCSA synthesis involved Williamson etherification and Knoevenagel condensation, followed by acid-catalyzed deprotection of the amine group. BPCSA·HI was obtained by stirring BPCSA in concentrated HI. FPCSA·HI was synthesized through demethylation, Williamson etherification, Knoevenagel condensation, and HI-catalyzed amine deprotection. 1H NMR and 13C NMR spectra of both BPCSA·HI and FPCSA·HI, along with their key intermediates, are presented in Supplementary Information (Figures S1–S18). Both AIEE LPs possess a rod-shaped cyanostilbene derivative framework with 1D bulkiness, allowing them to integrate into the 2D perovskite lattice. The propylamine group anchors [PbI_6_]^4−^ octahedrons through hydrogen bonding with I- ions, promoting co-crystallization with 2D PVSK layers.

Figure 2B,C confirm that both LPs exhibit stronger fluorescence in the solid phase (powder) than in solution, indicating AIEE activity. The synthesized FPCSA·HI powder emits at λ_max_ = 478 nm (Figure 2E), red-shifted relative to BPCSA·HI at 435 nm (Figure 2F). This shift results from the electron-accepting fluorine substitution in the donor (–OCH_3_)–π-acceptor (fluorine and CN groups) structure, stabilizing the polarized LUMO level. Both LP powders exhibit red-shifted PL excitation spectra compared to their absorption in DMSO, suggesting that solid-state emission arises from molecular planarization and energy-stabilizing dipole alignment, potentially through J-aggregation [44,48].

Single-layer quasi-2D AIEE_2_PbI_4_ PVSK films were fabricated via spin-coating, using precursor solutions prepared by mixing AIEE LPs and PbI_2_ in a 2:1 molar ratio within a DMF:DMSO (4:1 *v*/*v*) solvent system. Due to their structural compatibility, AIEE LPs facilitate participation within the quasi-2D perovskite lattice structure, yielding uniform films with a thickness of the BPCSA_2_PbI_4_ and FPCSA_2_PbI_4_ films of approximately 500 nm according to cross-sectional SEM (Figure S19A–D in the Supplementary Materials). However, the BPCSA_2_PbI_4_ film displays some visible pinholes, indicating slight non-uniformity. Simultaneously, OA_2_PbI_4_ films were fabricated under identical conditions to analyze the 2D perovskite characteristics of AIEE_2_PbI_4_ films and determine PbI_4_ valence band maximum (VBM) and conduction band minimum (CBM).

**Figure 3:**
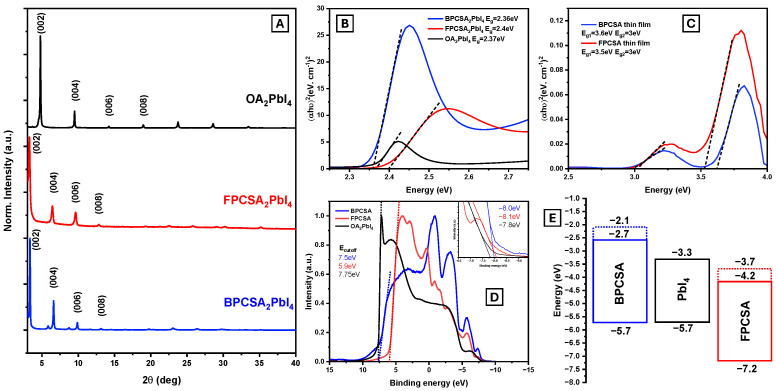
(**A**)—XRD patterns of BPCSA_2_PbI_4_, FPCSA_2_PbI_4_, and OA_2_PbI_4_ thin films. (**B**)—Optical bandgaps determined from Tauc plots derived from the UV-vis spectra of BPCSA_2_PbI_4_, FPCSA_2_PbI_4_, and OA_2_PbI_4_ thin films. The two absorption bands correspond to distinct aggregation modes. (**C**)—Tauc plots obtained from the UV-vis spectra of neat BPCSA and FPCSA thin films. (**D**)—UPS spectra of the respective films, with the inset highlighting the lower-energy band edges. (**E**)—Energy level alignment of AIEE LPs and 2D PVSK thin films, presenting a Type II band configuration between FPCSA and PbI_4_ and a Type I configuration between BPCSA and PbI_4_.

X-ray diffraction (XRD) analysis (Figure 3A) revealed distinct (002) diffraction peaks below 5° 2θ, with periodic (0,0,2n) reflections characteristic of layered quasi-2D Ruddlesden–Popper perovskite structures. The calculated out-of-plane layer spacings were 18.5 Å for OA_2_PbI_4_, 26.7 Å for BPCSA_2_PbI_4_, and 27.1 Å for FPCSA_2_PbI_4_. In the XRD pattern of BPCSA_2_PbI_4_, additional minor peaks at 2θ = 5.74° and 8.74° suggested the presence of a secondary n = 2 phase, consistent with the expected periodicity (estimated 2θ for (002) ≈ 2.9°). The full width at half maximum (FWHM) of the (002) peak was 0.11° for BPCSA_2_PbI_4_ and 0.36° for FPCSA_2_PbI_4_, indicating reduced crystallinity in FPCSA_2_PbI_4_ due to increased lattice distortion from the fluorinated ligand. Despite this, FPCSA_2_PbI_4_ exhibited remarkable ambient stability for over three months (see Figure S20, Supplementary Materials). While the XRD peak intensity decreased by approximately one-third, indicating partial degradation, the structural integrity remained largely preserved. Although long-term (multi-year) stability cannot yet be claimed, these results highlight the promising environmental resilience of FPCSA_2_PbI_4_ under ambient conditions. Nonetheless, for practical optoelectronic applications, encapsulation strategies will be necessary to ensure long-term device durability.

To assess energy level alignment, UV-vis absorption spectra of the 2D PVSK and AIEE LP thin films were analyzed. The AIEE_2_PbI_4_ exhibited an excitonic absorption feature near 500 nm (2.45 eV), while additional bands at ~350 nm (3.55 eV) were attributed to AIEE LP absorption, absent in the OA_2_PbI_4_ films (Figure S21 in Supplementary Materials). The optical bandgaps, determined from Tauc plots (Figure 3C), were 2.37 eV (OA_2_PbI_4_), 2.36 eV (BPCSA_2_PbI_4_), and 2.40 eV (FPCSA_2_PbI_4_). Given the minor differences, the OA_2_PbI_4_ PVSK bandgap was assumed equivalent to that of the PbI_4_ layer. The independent AIEE LP thin films, spin-coated from 5 mg/mL solutions, exhibited two distinct absorption bands at ~325 nm and ~380 nm, indicating different aggregation modes. These were considered in energy level alignment calculations.

Ultraviolet photoelectron spectroscopy (UPS) was performed on the AIEE LP and OA_2_PbI_4_ films to further refine energy level alignment. The VBM was determined by subtracting the He I UPS photon energy (21.22 eV) from the binding energy range of the sample, identified by two intersection points. The CBM and lowest unoccupied molecular orbital (LUMO) positions were estimated by subtracting the optical bandgap values (derived from the Tauc plots in Figure 3C,D) from the measured VBM values. The resulting energy band diagrams (Figure 3E) show that BPCSA exhibits a Type I alignment with PbI_4_, with a LUMO-CBM offset of ~0.6 eV for narrower bandgap aggregation and 1.2 eV for wider bandgap aggregation, alongside a HOMO-VBM offset of ~0 eV. Therefore, we can anticipate energy transfer from the AIEE LP to the PbI_4_ layer in BPCSA_2_PbI_4_. Conversely, as designed, FPCSA exhibits a Type II alignment with PbI_4_, with LUMO-CBM offsets of 1.1 eV (narrower bandgap) and 0.4 eV (wider bandgap), alongside a HOMO-VBM offset of 1.5 eV. The significant offset in both upper and lower energy levels in FPCSA_2_PbI_4_ inhibits energy transfer, minimizing charge transfer between hetero-excitons from organic and inorganic semiconductors. This promotes independent exciton recombination, as intended.

**Figure 4:**
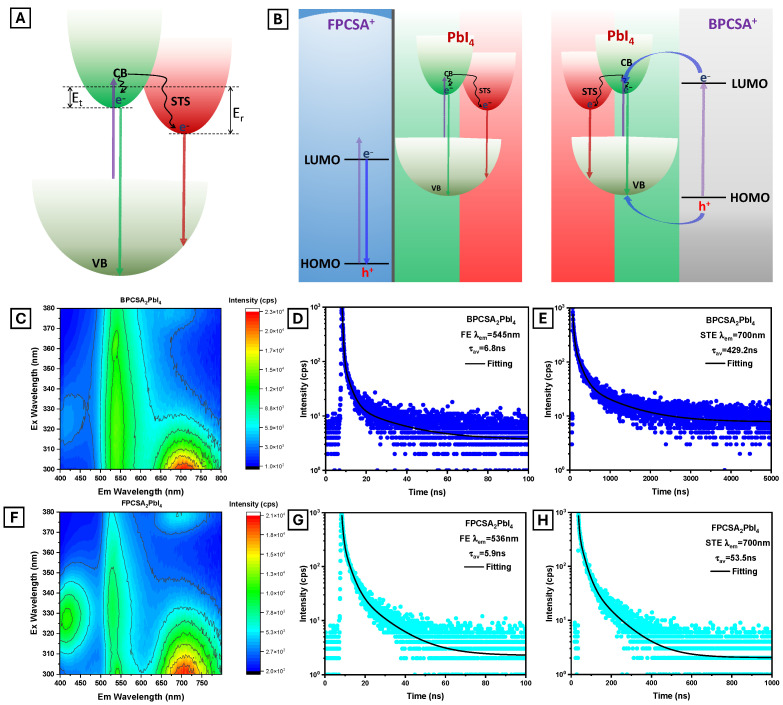
Schematic illustration of (**A**)—FE and STE emission of 2D PVSK and of (**B**)—energy transfer between FPCSA or BPCSA and PbI_4_ and the emission mechanism of the respective hybrids. (**C**,**F**)—Excitation-dependent PL emission color plots of BPCSA_2_PbI_4_ and FPCSA_2_PbI_4_, respectively. Time-resolved PL spectra of (**D**,**E**)—BPCSA_2_PbI_4_ FE and STE emission decay, respectively, and (**G**,**H**)—FPCSA_2_PbI_4_ FE and STE emission decay, respectively. A picosecond pulsed 375 nm laser was used as an excitation source.

We measured and analyzed the excitation wavelength-dependent PL spectra and time-resolved PL to assess the photoluminescence (PL) characteristics of AIEE_2_PbI_4_ PVSK thin films. Figure 3A illustrates the FE and STE emission mechanisms in 2D PVSK, while Figure 3B represents the proposed mechanisms for multiple emission bands from AIEE_2_PbI_4_ PVSKs. As shown in Figure 4A, an energy barrier (E_t_) exists for transferring the excited electron to the self-trapped state (STS), which can be reached through higher energy excitation, thermal activation, or tunneling pathways [49]. We screened the excitation wavelength-dependent PL spectra from 300 to 380 nm to obtain the STE and AIEE LP emissions simultaneously. As a control, we recorded the PL of the conventional quasi-2D PVSK, OA_2_PbI_4_, which exhibited a typical narrow single emission band at 520 nm upon 315 nm excitation (Figure S22 in the Supplementary Materials). This indicates negligible lattice distortion due to the van der Waals force-driven assembly of flexible octadecyl ammonium (OA) cations. However, BPCSA_2_PbI_4_ displays two dominant emission peaks: a narrow band at approximately 545 nm, attributed to the FE emission of the 2D PVSK phase, and a broadband at around 700 nm, potentially arising from self-trapped exciton (STE) emission (Figure 4C). The broad and significantly Stokes-shifted emission upon 330 nm or shorter wavelength excitation provides evidence of STE. In previously reported BPCSA_2_MA_n−1_Pb_n_I_3n+1_, BPCSA absorbs light and transfers its excitation energy to the 2D PVSK layers, which emit the characteristic 2D PVSK PL without STE emission [47]. Consequently, the single-layer and strong π-π stacking assembly of rigid AIEE cations can induce significant lattice distortion and generate STS. However, the energy transfer from BPCSA to the PbI_4_ layer in the Type I energy level alignments of BPCSA_2_PbI_4_ results in the faint intensity of BPCSA emission at 410 nm (Figure 4B,C). The FE lifetime is typically sub-nanosecond, while the STE lifetime can extend to several microseconds [50]. Figure 4D,E show the PL lifetimes of BPCSA_2_PbI_4_ for 545 nm and 700 nm emissions, providing evidence of the corresponding emission mechanisms. The resulting 6.8 ns and 429 ns lifetimes correlate well with the FE and STE emission mechanisms.

As mentioned, FPCSA was designed to prevent energy transfer between the AIEE LP and 2D PVSK phases. The shift of HOMO/LUMO levels in the highly electronegative fluorine-substituted AIEE LP enables a Type II or Type III band alignment with the PbI_4_ layer, and we successfully obtained a Type II band alignment with FPCSA (Figure 3E). Type II alignment prevents energy transfer but can induce charge transfer, reducing exciton recombination. To efficiently suppress charge transfer between two semiconductors, such as an organic dye and a perovskite layer, the energy offset between their band edges (i.e., the HOMO/LUMO of the organic material and the VBM/CBM of the inorganic semiconductor) must exceed the exciton binding energy or the thermal energy at room temperature [31]. Research on organic photovoltaic cells has demonstrated that a driving force (energy offset) of at least 0.3 eV is necessary for efficient charge separation, and increasing this offset can further inhibit undesired charge recombination processes [51]. As shown in Figure 3E, the 0.4 to 1.1 eV offset between the CBM and LUMO and the 1.5 eV offset between the VBM and HOMO effectively suppress charge transfer between FPCSA and the 2D PVSK phase. Consequently, excitons generated in either the FPCSA or the 2D PVSK phase remain confined to their respective layers, resulting in independent PL from both components (Figure 4B).

The PL emission/excitation contour map (Figure 4F) illustrates the triple-band emission of FPCSA_2_PbI_4_ when excited at 310 to 335 nm. The shortest-wavelength emission band, appearing at 410 nm, arises from the recombination of FPCSA excitons. This band is significantly blue-shifted compared to its emission in powder form, which can be attributed to the broader bandgap aggregation of FPCSAs during the assembly of the 2D PVSK lattice. The excitation wavelength for the 410 nm emission aligns well with the absorption spectrum of FPCSA (Figure 2F). The FPCSA emission from FPCSA_2_PbI_4_ indicates that the FPCSA exciton is isolated from the PVSK layer. The second peak across all excitation wavelength ranges corresponds to the FE emission of the PVSK. Additionally, a broad and intense emission band at 700 nm is observed upon excitation at 340 nm or shorter wavelengths, attributed to STE emission. The PL lifetimes for FPCSA_2_PbI_4_ are 5.9 ns for FE emission and 53.5 ns for STE emission (Figure 4G,H). The significantly shorter STE emission lifetime of FPCSA_2_PbI_4_ compared to BPCSA_2_PbI_4_ is due to differing energies for reverse electron transfer (E_r_) (see Figure 4A). Given the similar STE emission wavelengths for both PVSKs, the energy gap between the STS and VBM of the 2D PVSKs is comparable. However, the longest excitation wavelength for STE emission is 10 nm shorter for BPCSA_2_PbI_4_ than for FPCSA_2_PbI_4_. Therefore, E_r_ is lower for FPCSA_2_PbI_4_, enhancing the probability of de-trapping from STS and emissions as FE.

**Figure 5:**
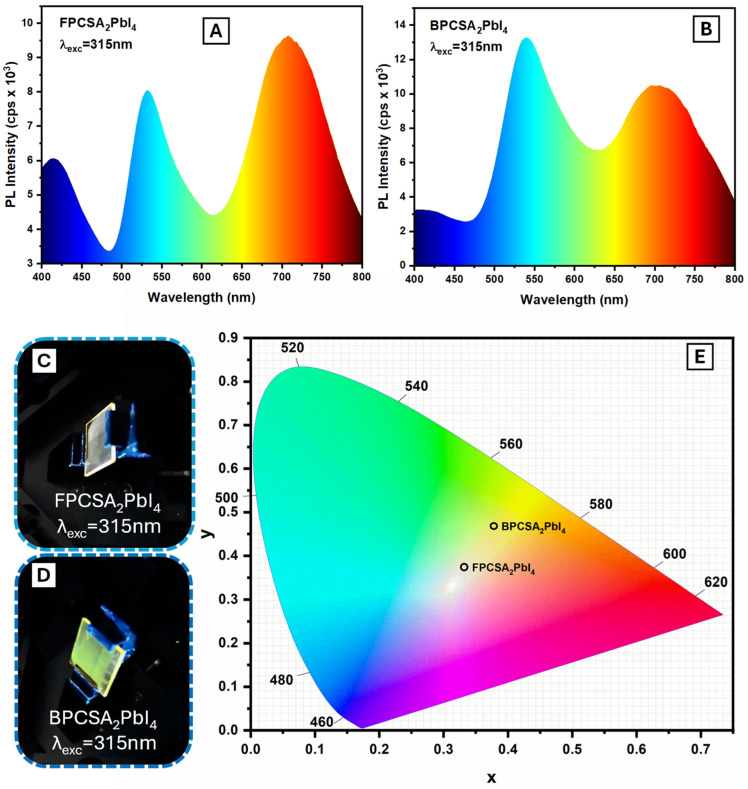
PL emission under 315 nm excitation of (**A**)—FPCSA_2_PbI_4_ and (**B**)—BPCSA_2_PbI_4_. Photographs of (**C**)—FPCSA_2_PbI_4_ and (**D**)—BPCSA_2_PbI_4_ thin films under 315 excitation. (**E**)—CIE coordinates of FPCSA_2_PbI_4_ (0.33, 0.38) and BPCSA_2_PbI_4_ (0.38, 0.47).

The emission spectra for FPCSA_2_PbI_4_ and BPCSA_2_PbI_4_ upon 315 nm excitation are presented in Figure 5A,B, and their luminescence photographs are displayed in Figure 5C,D. As discussed, FPCSA_2_PbI_4_ exhibits a triple emission band at ambient temperature. The concerted triple-band emission of FPCSA_2_PbI_4_ produces warm white luminescence (Figure 5C) with CIE coordinates of (0.33, 0.38) (Figure 5E). This broad-spectrum emission results from the combined contributions of three distinct bands: blue emission from FPCSA, green emission from 2D PVSK free excitons (FEs), and red emission from self-trapped excitons (STEs). In contrast, BPCSA_2_PbI_4_ also displays prominent double-band (red and green) emission (Figure 4B), but its blue emission from BPCSA is significantly quenched due to charge transfer. This quenching leads to a dominant contribution from the green and red emissions, resulting in a yellowish emission (Figure 5D) with CIE coordinates of (0.38, 0.47) (Figure 5E).

## 3. Materials and Methods

### 3.1. Materials

4-Hydroxybenzaldehyde (98%) and 3-(Boc-amino)propyl bromide (95%) were purchased from AKScientific (Union City, CA, USA). 4′-methoxy-[1,1′-biphenyl]-4-carbaldehyde (96%) and 2,3,4,5,6-pentafluorophenyl acetonitrile (98%) were purchased from Thermo Fisher Scientific (Waltham, MA, USA). Trifluoroacetic acid (99%), tetrabutylammonium hydroxide solution (1.0 M in methanol), potassium carbonate (anhydrous, ACS reagent, ≥99%), sodium bicarbonate (ACS reagent, ≥99.7%), sodium hydroxide (98%), boron tribromide (99.9%), Dichloromethane (98%), Acetone (99.5%), and tert-Butanol (99.3%) were purchased from Merck (Rahway, NJ, USA). Lead (II) iodide (PbI298%), Octylamine (OA, 99%), hydroiodic acid (57wt% in water), dimethyl formamide (DMF, anhydrous 99%), and dimethyl sulfoxide (DMSO, anhydrous 99%) were purchased from Merck. Indium-doped and Fluoride-doped tin oxide (ITO and FTO, respectively) substrates were purchased from OPV Tech (Yingkou, Liaoning, China). All chemicals were used as received without further purification.

### 3.2. Synthesis of BPCSA

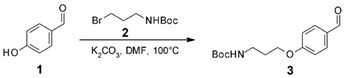



***tert*-Butyl (3-(4-formylphenoxy)propyl)carbamate (3).** A mixture of tert-butyl (3-bromopropyl)carbamate (**2**) (4.890 g, 20.5 mmol, 1 eq.), 4-hydroxybenzaldehyde (3.013 g, 24.6 mmol, 1.2 eq.), and potassium carbonate (2.840 g, 20.5 mmol, 1 eq.) in 30 mL of DMF was stirred for 4 h at room temperature and 30 min at 100 °C. The reaction mixture was diluted with water and extracted with MTBE. The combined organic phases were washed twice with water, followed by washing twice with 5% NaOH solution and water. Afterward, the organic phase was dried with Na_2_SO_4_ and filtered; then, the solvent was evaporated under reduced pressure, and the residue was dried in vacuum to afford pure product **3** as yellowish crystals. Yield 4.684 g (82%). ^1^H NMR (500 MHz, CDCl_3_) δ = 1.43 (s, 9 H), 1.93–2.07 (m, 2 H), 3.27–3.42 (m, 2 H), 4.09 (t, *J =* 6.01 Hz, 2 H), 4.74–4.88 (m, 1 H), 6.98 (d, *J =* 8.59 Hz, 2 H), 7.82 (d, *J =* 8.59 Hz, 2 H), 9.87 (s, 4 H) ppm. ^13^C NMR (126 MHz, CDCl_3_) δ = 28.3, 29.4, 37.7, 66.0, 79.3, 114.7, 129.9, 132.0, 156.0, 163.8, 190.8 ppm.

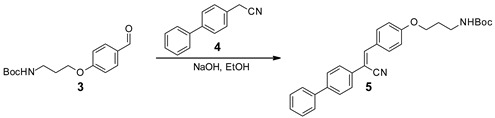



***tert*-Butyl(3-(4-(2-([1,1′-biphenyl]-4-yl)-2-cyanovinyl)phenoxy)propyl)carbamate (5).** A solution of compound **3** (4.679 g, 16.7 mmol, 1.00 eq.), 2-([1,1′-biphenyl]-4-yl)acetonitrile (**4**) (3.403 g, 17.6 mmol, 1.05 eq.), and NaOH (1.00 g, 25.2 mmol, 1.5 eq.) in ethanol (150 mL) was stirred at room temperature for 21 h. Then, the precipitated product was filtrated off, washed twice with cold ethanol, washed twice with diethyl ether, and dried. Yield 7.103 g (93%). ^1^H NMR (500 MHz, CDCl_3_) δ = 1.46 (s, 9 H), 1.99–2.08 (m, 2 H), 3.36 (d, *J =* 6.30 Hz, 2 H), 4.10 (t, *J =* 6.01 Hz, 2 H), 6.99 (d, *J =* 8.59 Hz, 2 H), 7.40 (d, *J =* 6.87 Hz, 1 H), 7.48 (t, *J =* 7.45 Hz, 2 H), 7.52 (s, 1 H), 7.64 (d, *J =* 6.87 Hz, 2 H), 7.66–7.77 (m, 4 H), 7.91 (d, *J =* 8.59 Hz, 2 H) ppm. ^13^C NMR (126 MHz, CDCl_3_) δ = 28.4, 29.4, 37.8, 65.9, 79.3, 108.2, 114.8, 118.5, 126.1, 126.6, 127.0, 127.6, 127.7, 128.9, 131.2, 133.7, 140.0, 141.5, 141.5, 156.0, 160.6 ppm.

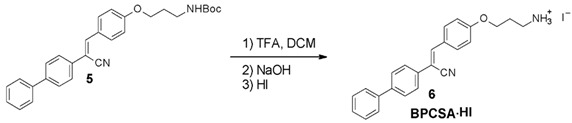



**3-(4-(2-([1,1′-biphenyl]-4-yl)-2-cyanovinyl)phenoxy)propan-1-aminium 2,2,2-trifluoroacetate (BPCSA·TFA).** To a solution of compound **5** (7.103 g, 15.6 mmol in DCM) (50 mL), trifluoroacetic acid (10 mL) was added, and the mixture was stirred at room temperature for 18 h. Afterward, the volatiles were evaporated under reduced pressure; the obtained solid product was washed with diethyl ether, filtrated, and dried. Yield 7.341 g (100%); pale-yellow solid. ^1^H NMR (500 MHz, DMSO-d6) δ = 2.04 (quin, *J =* 6.69 Hz, 2 H), 2.99 (br. s., 2 H), 4.16 (t, *J =* 6.09 Hz, 2 H), 7.10–7.16 (m, 2 H), 7.37–7.44 (m, 1 H), 7.46–7.54 (m, 2 H), 7.71–7.76 (m, 2 H), 7.80–7.91 (m, 7 H), 7.99 (d, *J =* 8.88 Hz, 2 H), 8.05 (s, 4 H) ppm. ^13^C NMR (126 MHz, DMSO-d6) δ = 26.8, 36.3, 64.9, 106.8, 115.0, 118.4, 126.1, 126.5, 126.7, 127.3, 127.9, 129.1, 131.2, 133.2, 139.1, 140.4, 142.2, 158.4, 158.7, 160.3 ppm.

**BPCSA (6). BPCSA∙TFA** (7.321 g, 15.6 mmol) was converted into a base by suspending it in water (400 mL), followed by the addition of 5% sodium hydroxide solution (100 mL). The mixture was vigorously stirred for 15 min; afterward, the product was extracted into DCM. The combined organic phases were dried with Na_2_SO_4_ and filtered; then, the solvent was evaporated under reduced pressure to afford the desired product as a pale-yellow solid. Yield 4.998 g (90%). ^1^H NMR (500 MHz, DMSO-d6) δ = 1.81 (quin, *J =* 6.59 Hz, 2 H), 2.70 (t, *J =* 6.59 Hz, 2 H), 4.13 (t, *J =* 6.30 Hz, 2 H), 7.11 (d, *J =* 8.59 Hz, 2 H), 7.36–7.44 (m, 1 H), 7.50 (t, *J =* 7.73 Hz, 2 H), 7.74 (d, *J =* 7.45 Hz, 2 H), 7.82 (s, 4 H), 7.97 (d, *J =* 8.59 Hz, 2 H), 8.03 (s, 1 H) ppm. ^13^C NMR (126 MHz, DMSO-d6) δ = 32.6, 38.3, 65.9, 106.4, 115.0, 118.5, 126.0, 126.1, 126.7, 127.3, 127.9, 129.1, 131.3, 133.2, 139.1, 140.3, 142.3, 160.8 ppm.

**BPCSA·HI** (**6**) was obtained by stirring the base BPCSA (0.893 g, 2.5 mmol) in 10 mL HI (57%) for 3 h. The salt was centrifuged and washed with water three times. Then, it was dissolved in acetonitrile and evaporated under reduced pressure to remove the residual water. Yield 1.141 g (95%). ^1^H NMR (500 MHz, DMSO-d6) δ = 8.06 (s, 1 H), 7.99 (d, *J* = 9.0 Hz, 2 H), 7.83 (s, 4 H), 7.74 (d, *J* = 7.3 Hz, 2 H), 7.50 (t, *J* = 7.7 Hz, 2 H), 7.43–7.38 (m, 1 H), 7.13 (d, *J* = 8.9 Hz, 2 H), 4.16 (t, *J* = 6.0 Hz, 2 H), 2.96 (t, *J* = 7.3 Hz, 2 H), 2.02 (quin, *J* = 6.7 Hz, 2 H) ppm. ^13^C NMR (126 MHz, DMSO-d6) δ = 27.5, 36.9, 65.4, 107.2, 115.4, 115.6, 118.9, 126.6, 127.0, 127.2, 127.8, 129.6, 131.7, 133.7, 139.5, 140.9, 142.8, 160.7 ppm.

### 3.3. Synthesis of FPCSA

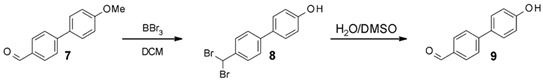



**4′-Hydroxy-[1,1′-biphenyl]-4-carbaldehyde (9).** To a solution of aldehyde **7** (5.052 g, 23.6 mmol, 1 eq.) in DCM (40 mL) under argon atmosphere, boron tribromide (6.7 mL, 70.7 mmol, 3 eq.) was added, and the mixture was stirred for 4 h at room temperature. Then, the reaction mixture was poured into an ice–water mixture, stirred for 15 min, and subsequently extracted with DCM. Then, the combined organic phases were washed three times with a sodium bicarbonate solution and twice with water, dried with Na_2_SO_4_, filtered, and evaporated under reduced pressure to afford crude **8**, which was used in the next step.

The obtained solid product (**8**) was dissolved in a DMSO–water mixture (56 mL/10 mL, respectively), and the solution was stirred overnight at room temperature. Subsequently, the reaction mixture was diluted with water and extracted with ethyl acetate. The combined organic phases were washed three times with water, dried with Na_2_SO_4_, filtered, and evaporated under reduced pressure; then, the residue was dried in vacuum to afford pure **9**. Yield 7.620 g (89%). ^1^H NMR (500 MHz, DMSO-d6) δ = 6.89 (d, *J =* 9.17 Hz, 2 H), 7.63 (d, *J =* 8.02 Hz, 2 H), 7.82 (s, 2 H), 7.94 (s, 2 H), 9.80 (s, 1 H), 10.01 (s, 1 H) ppm. ^13^C NMR (126 MHz, DMSO-d6) δ = 116.0, 126.4, 128.4, 129.3, 130.2, 134.2, 146.0, 158.3, 192.6 ppm.

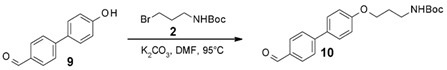



***tert*-Butyl (3-((4′-formyl-[1,1′-biphenyl]-4-yl)oxy)propyl)carbamate (10).** A mixture of 3-(Boc-amino)propyl bromide (**2**) (4.933 g, 20.7 mmol, 0.98 equiv.), compound **9** (4.191 g, 21.1 mmol, 1.00 equiv.), and K_2_CO_3_ (8.775 g, 63.5 mmol, 3.00 equiv.) in 52 mL of DMF was stirred at 95 °C for 23 h. Subsequently, the reaction mixture was cooled down, diluted with water, and extracted with MTBE. The combined organic phases were washed with water, followed by washing with 5% NaOH solution and water, drying with Na_2_SO_4_, filtering, and evaporation under reduced pressure. Then, the residue was dried in vacuum to afford pure **10**. Yield 6.769 g (90%). ^1^H NMR (500 MHz, CDCl_3_) δ = 1.46 (s, 9 H), 1.98–2.07 (m, 2 H), 3.36 (q, *J =* 5.70 Hz, 2 H), 4.09 (t, *J =* 5.73 Hz, 2 H), 4.79 (br. s., 1 H), 7.00 (d, *J =* 8.59 Hz, 2 H), 7.57–7.62 (m, 2 H), 7.72 (d, *J =* 8.02 Hz, 2 H), 7.93 (d, *J =* 8.02 Hz, 2 H), 10.04 (s, 1 H) ppm. ^13^C NMR (126 MHz, CDCl_3_) δ = 28.4, 29.5, 37.9, 65.8, 79.3, 114.9, 127.0, 128.5, 130.3, 132.1, 134.6, 146.7, 156.0, 159.3, 191.9 ppm.

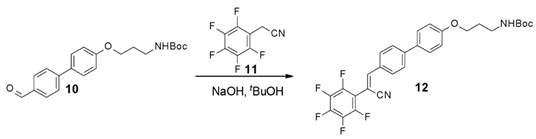



***tert*-Butyl (3-((4′-(2-cyano-2-(perfluorophenyl)vinyl)-[1,1′-biphenyl]-4-yl)oxy)propyl)carbamate (12).** A mixture of compound **10** (5.680 g, 16.0 mmol, 1.00 eq.), pentafluorophenylacetonitrile (**11**) (3.475 g, 16.78 mmol, 1.05 eq.), and NaOH (2.006 g, 47.9 mmol, 3.00 eq.) in 70 mL *^t^*BuOH was stirred at room temperature for 25 h. Then, the reaction mixture was diluted with water and extracted with MTBE. The combined organic phases were washed with water, dried with Na_2_SO_4_, filtered, and evaporated under reduced pressure. Then, the residue was dissolved in DCM, and the product was precipitated from the solution by adding hexane. The precipitate was filtrated out, washed with diethyl ether, and dried. Yield 6.208 g (71%). ^1^H NMR (500 MHz, DMSO-*d*_6_) δ = 1.38 (s, 9 H), 1.85 (quin, *J =* 6.60 Hz, 2 H), 3.10 (q, *J =* 6.60 Hz, 2 H), 4.04 (t, *J =* 6.23 Hz, 2 H), 6.94 (t, *J =* 5.58 Hz, 1 H), 7.05 (d, *J =* 8.74 Hz, 2 H), 7.75 (d, *J =* 8.73 Hz, 2 H), 7.87–7.91 (m, 3 H), 8.03 (d, *J =* 8.45 Hz, 2 H) ppm. ^13^C NMR (126 MHz, DMSO-d6) δ = 28.8, 29.7, 37.4, 65.9, 78.0, 93.7, 115.6, 116.9, 127.2, 128.6, 130.8, 131.0, 131.3, 143.8, 153.0, 156.2, 159.6 ppm.

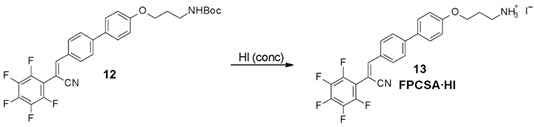



**FPCSA·HI** (**13**) A suspension of compound **12** (2.008 g, 3.69 mmol) in HI (57% solution water, 20 mL) was stirred at room temperature for 4 h. The precipitate was centrifuged and washed three times with water. Then, the wet residue was dissolved in acetonitrile, evaporated, and dried in vacuum. Yield 2.049 g (97%). ^1^H NMR (500 MHz, DMSO-*d*_6_) 2.04 (quin, *J =* 6.80 Hz, 2 H), 3.00 (sxt, *J =* 6.20 Hz, 2 H), 4.14 (t, *J =* 6.01 Hz, 2 H), 7.08 (d, *J =* 8.88 Hz, 2 H), 7.67–7.75 (m, 3 H), 7.75–7.80 (m, 2 H), 7.88 (s, 1 H), 7.88–8.07 (m, 4 H) ppm. ^13^C NMR (126 MHz, DMSO-d6) δ = 26.8, 36.4, 64.7, 93.2, 115.1, 116.3, 126.7, 128.2, 130.3, 130.6, 131.0, 143.2, 152.5, 158.8 ppm.

### 3.4. Characterization

XRD measurements were conducted using a Rigaku Smartlab X-ray diffractometer (Rigaku, Tokyo, Japan) with Cu Kα (λ = 1.54 Å) radiation. The crystallographic characteristics of the AIE-dye-incorporated 2D PVSK films were analyzed as synthesized. The XRD spectra were analyzed using SmartLab studio software version 4.4. The UV-vis absorbance spectra of perovskite films were recorded using a PerkinElmer Lambda 1050 spectrometer (Waltham, MA, USA). The absorption spectrum of the clean substrate was first measured and then subtracted from the spectrum of the sample. Steady-state photoluminescence spectra of the perovskite films were recorded by a fluorescent spectrophotometer (FLS1000, Edinburgh Instruments, Edinburgh, UK) with a 200 W Xe lamp as an excitation source. TCSPC measurements were performed using an Edinburgh Instruments spectrometer (FLS1000) with a 375 nm pulsed laser. Ultraviolet photoelectron spectroscopy (UPS) was conducted using a NEXSA (Thermo Scientific, Waltham, MA, USA) spectrometer under ultrahigh vacuum conditions (~1 × 10^−10^ mbar) with a monochromatic UV source providing photons at 21.22 eV. An external bias voltage of −10 V was applied to the perovskite films. The UPS spectra were analyzed using Avantage software version 5.9931.0.6755.

## 4. Conclusions

In this study, two AIEE LP cations, BPCSA and FPCSA, were successfully synthesized and integrated into 2D PVSK structures to achieve a white luminescence, combining isolated RGB triple-band emission in a single-phase material. The synthesized cations demonstrated structural compatibility with the perovskite lattice, facilitating their incorporation into perovskite films through spin-coating. XRD analysis confirmed the formation of layered crystalline structures, while UV-vis spectroscopy revealed characteristic excitonic features and energy band misalignments essential for achieving multiband emission.

BPCSA_2_PbI_4_ and FPCSA_2_PbI_4_ films exhibited distinct emission behaviors due to differences in band alignment and charge transfer dynamics. BPCSA_2_PbI_4_ followed a Type I band alignment, leading to energy transfer and the quenching of its blue emission from BPCSA, resulting in a dominant yellowish emission. In contrast, FPCSA_2_PbI_4_ adopted a Type II alignment, effectively suppressing charge transfer and enabling independent emissions from both the AIEE dye and the 2D perovskite layer. This resulted in triple-band emission spanning blue, green, and red wavelengths, leading to a warm white-light output under 315 nm excitation. The significant reduction in self-trapped exciton (STE) emission lifetime in FPCSA_2_PbI_4_, compared to BPCSA_2_PbI_4_, suggested a higher degree of octahedral distortion within the perovskite framework. Additionally, the observed tradeoff between 410 nm and 700 nm emission intensities highlighted the influence of the excitation wavelength on the spectral distribution.

Overall, this work demonstrates that careful molecular design and energy level engineering of AIEE ligands enable tunable light emission properties in hybrid 2D perovskite systems. The findings provide valuable insights for the development of multi-color and white-light-emitting perovskite materials, with potential applications in optoelectronic and lighting technologies. While this material may not be ideal for display applications requiring narrowband emission, we now clarify that FPCSA_2_PbI_4_ is more suited for applications such as ambient or architectural lighting, solid-state white-light sources, and decorative illumination, where warm white light and a broad spectrum are desired.

## Data Availability

The original contributions presented in this study are included in this article/the Supplementary Materials. Further inquiries can be directed to the corresponding author(s).

## Supplementary Materials

The following supporting information can be downloaded at https://www.mdpi.com/article/10.3390/ijms26115054/s1.
