# Supplementary material for: Triple-Band Warm White-Light Emission from Type II Band-Aligned Aggregation-Induced Enhanced Emission Organic Cation-Incorporated Two-Dimensional Lead Iodide Perovskite
Source: Int J Mol Sci. 2025 May 24;26(11):5054. doi: 10.3390/ijms26115054 (PMC12155297; doi:10.3390/ijms26115054)
Supplement: Supplementary file 1 [file ijms-26-05054-s001.zip › ijms-3598256-supplementary.pdf]

Supplementary Materials

Triple-Band Warm White-Light Emission from Type II Band-Aligned Aggregation-Induced Enhanced Emission Organic Cation-Incorporated Two-Dimensional Lead Iodide Perovskite

Almaz R. Beisenbayev [1,†], Igor Ivanov-Prianichnikov [2,†], Anatoly Peshkov [1], Tangsulu Adil [1], Davit Hayrapetyan [2,*] and Chang-Keun Lim [1,*]

[1] Department of Chemical and Materials Engineering, School of Engineering and Digital Sciences, Nazarbayev University, Astana 010000, Kazakhstan

[2] Department of Chemistry, School of Sciences and Humanities, Nazarbayev University, Astana 010000, Kazakhstan

[†] These authors contributed equally to this work.

* Correspondence: davit.hayrapetyan@nu.edu.kz (D.H.), changkeun.lim@nu.edu.kz (C.-K.L.)

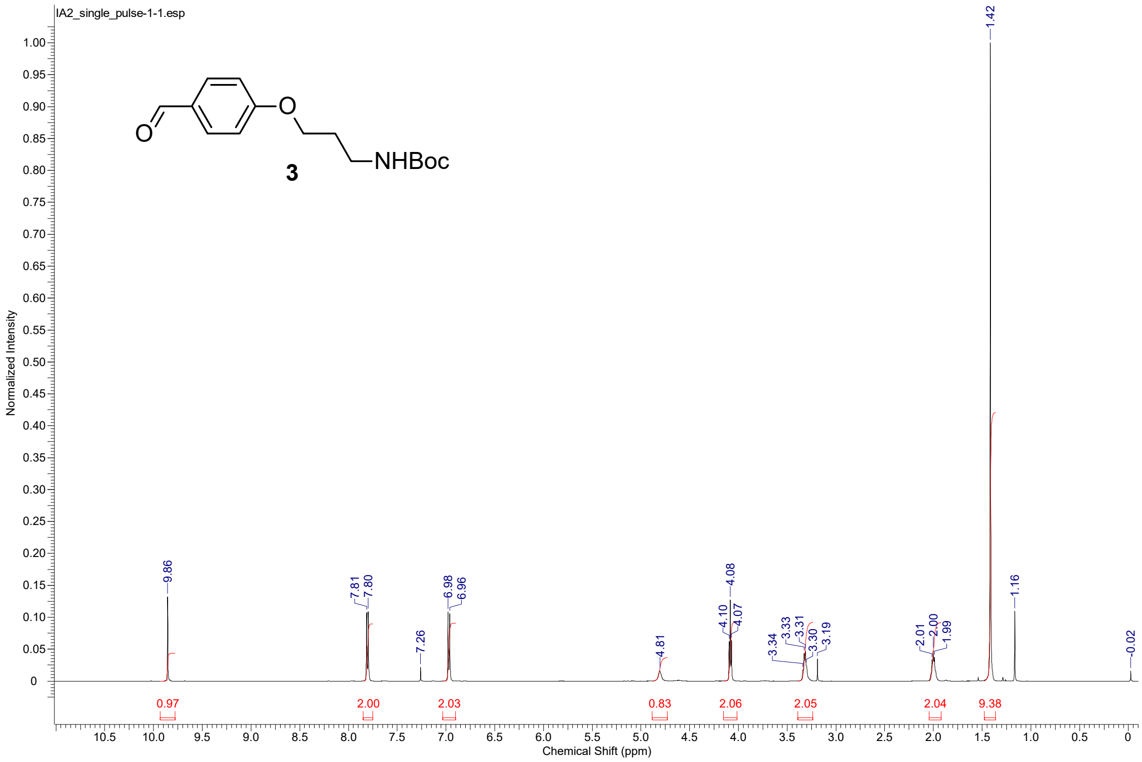

Figure S1. ^{1}H NMR spectra of compound **3**.

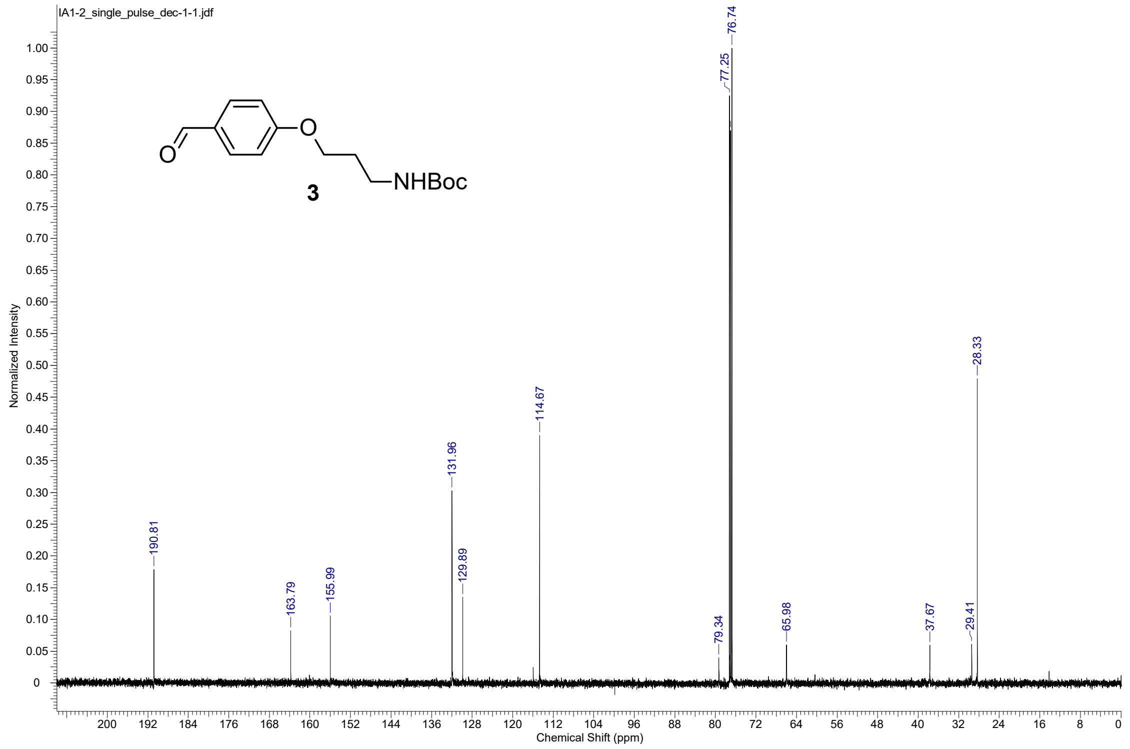

Figure S2. ^{13}C NMR spectra of compound **3**.

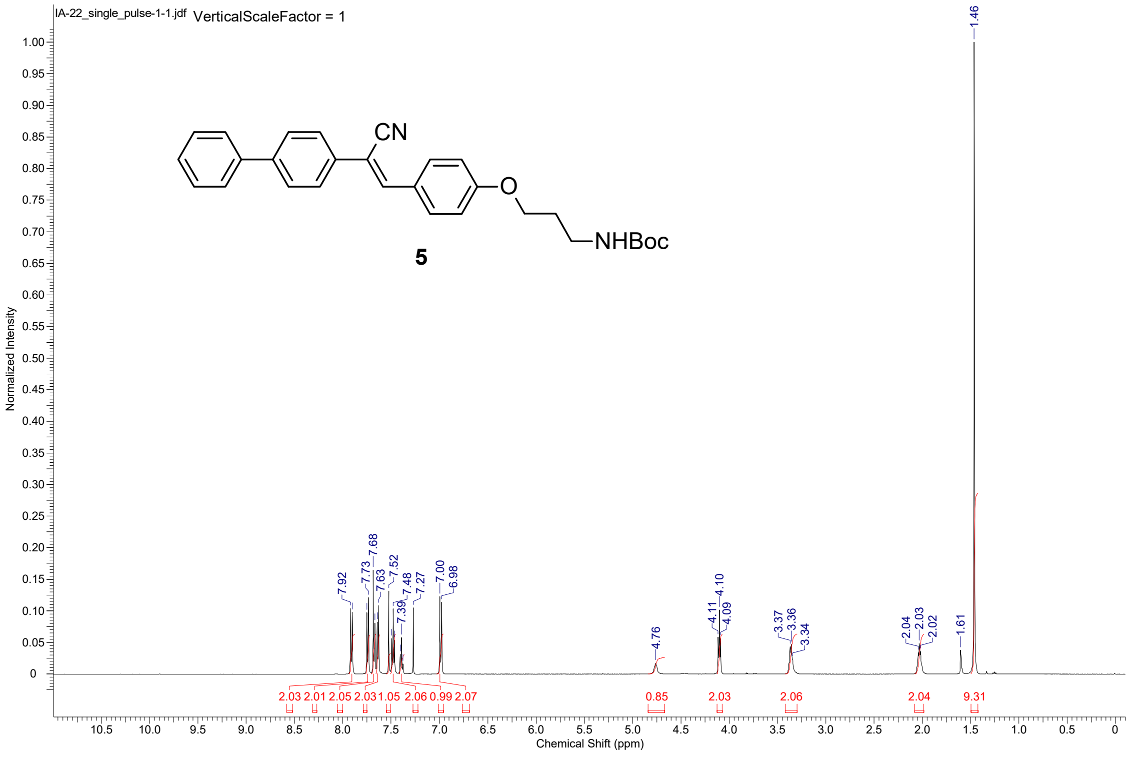

Figure S3. ^{1}H NMR spectra of compound **5**.

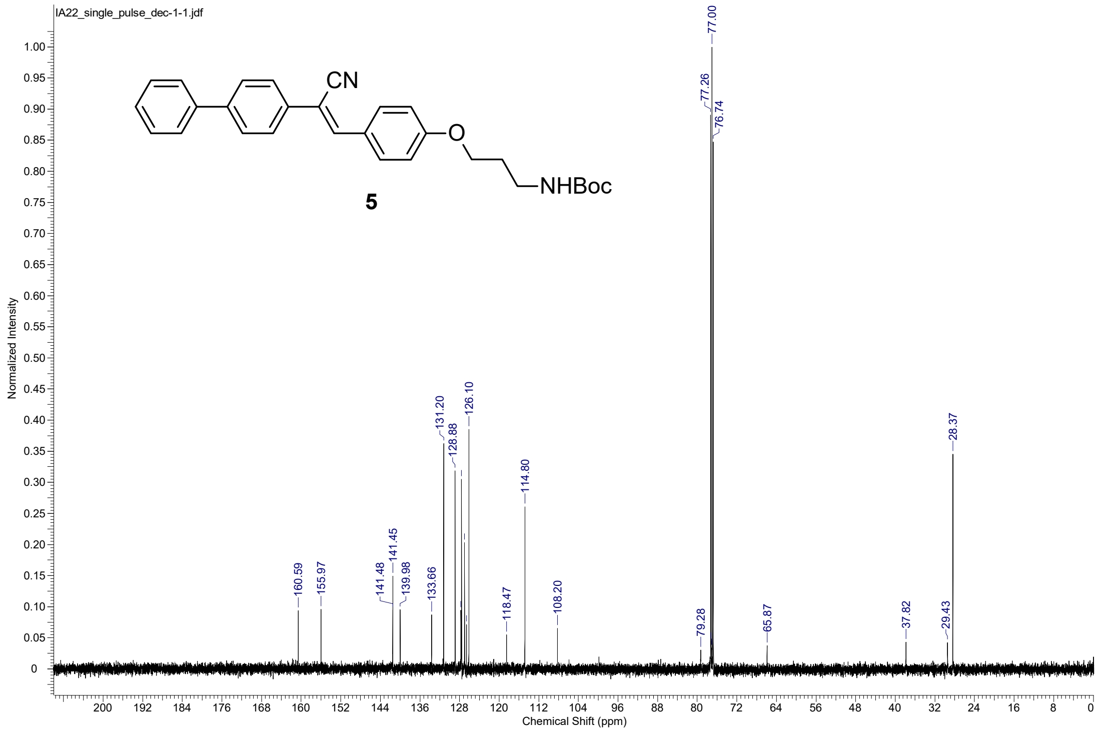

Figure S4. ^{13}C NMR spectra of compound **5**.

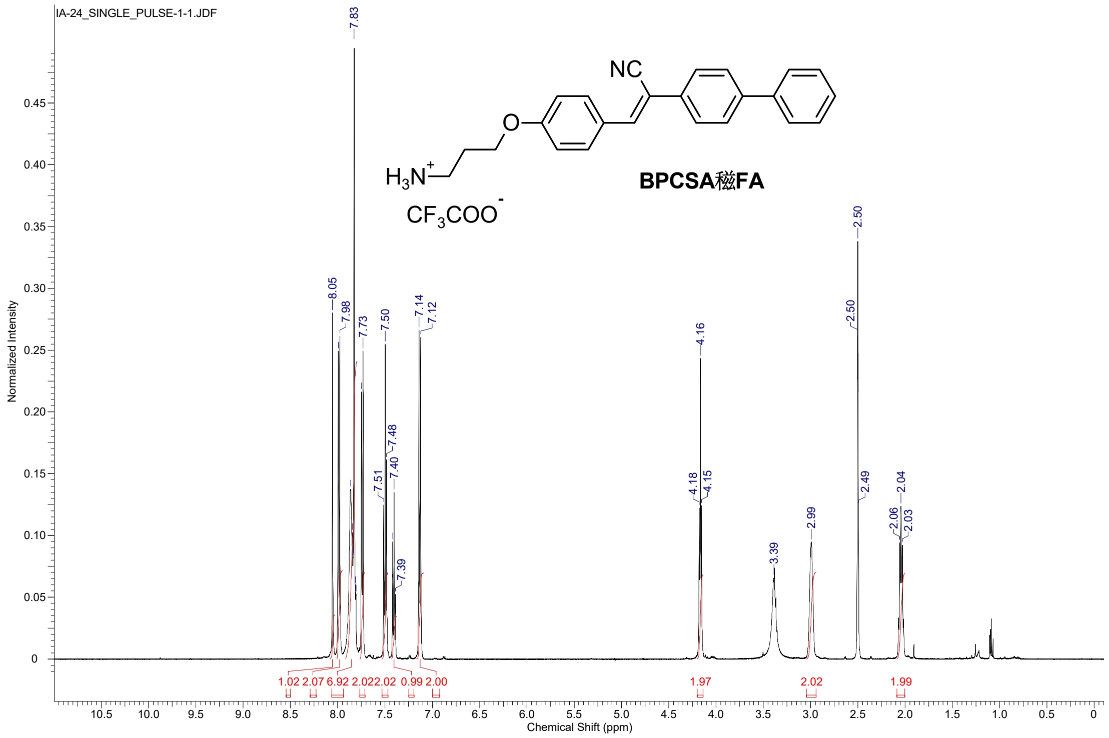

Figure S5. ^{1}H NMR spectra of TFA salt of BPCSA.

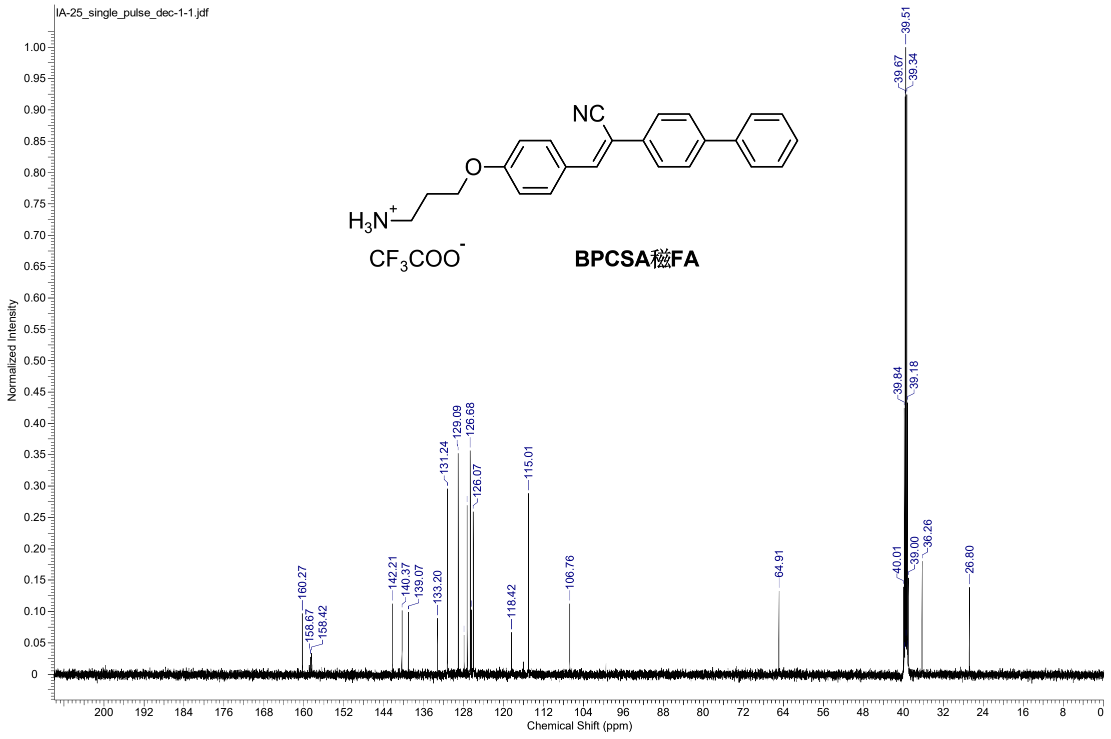

Figure S6. ^{13}C NMR spectra of TFA salt of BPCSA.

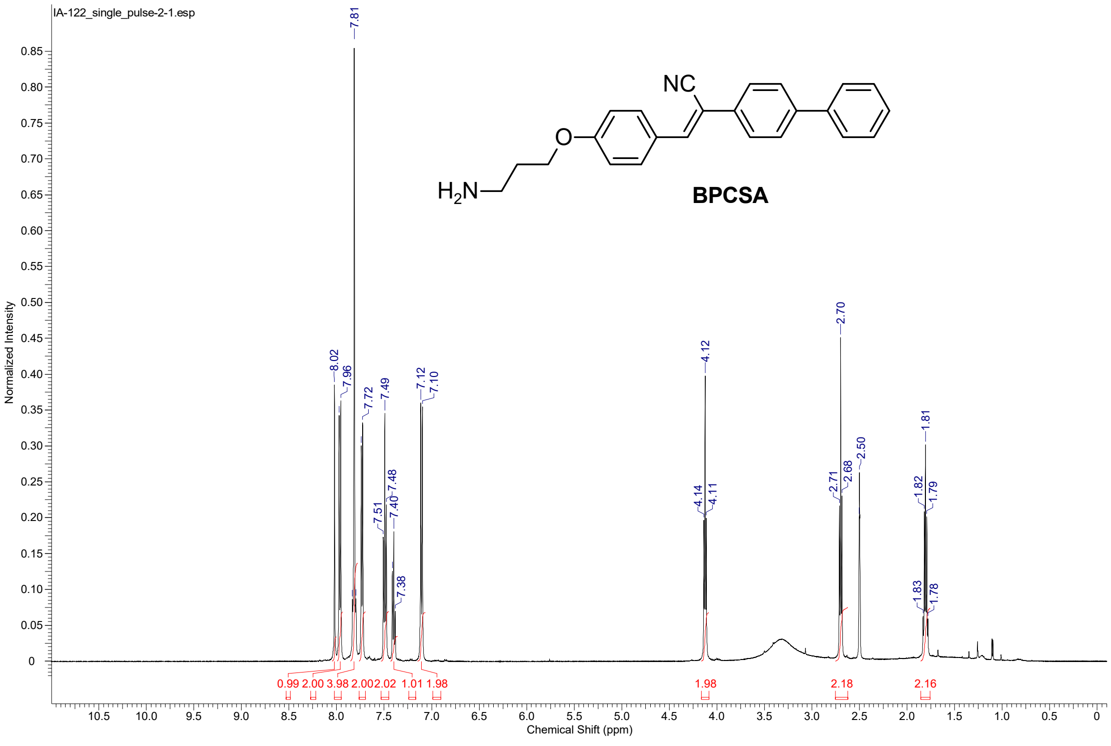

Figure S7. ^{1}H NMR spectra of BPCSA base.

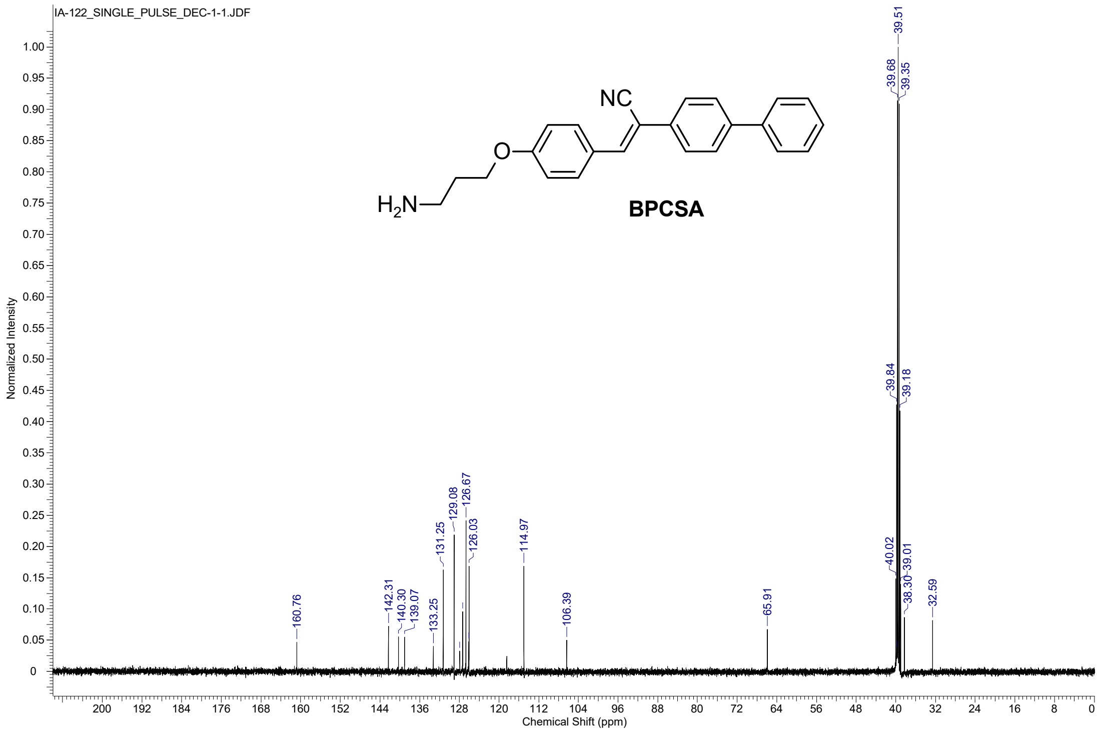

Figure S8. ^{13}C NMR spectra of BPCSA base.

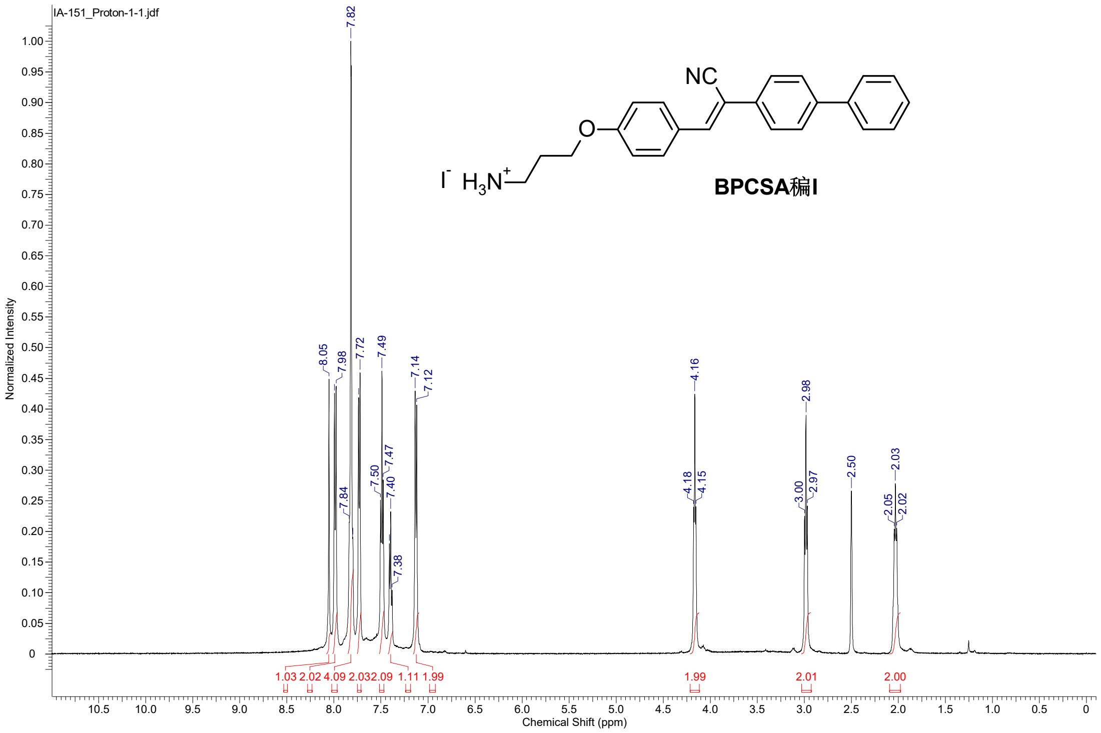

Figure S9. ^{1}H NMR spectra of compound 6.

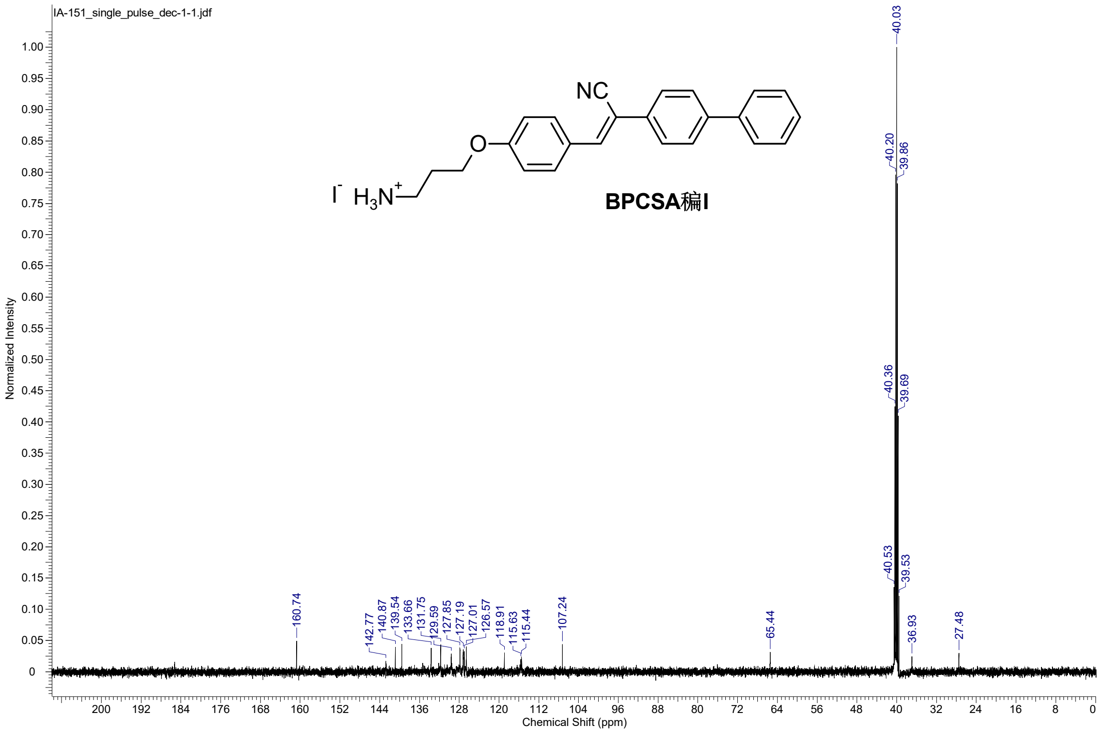

Figure S10. ^{13}C NMR spectra of compound 6.

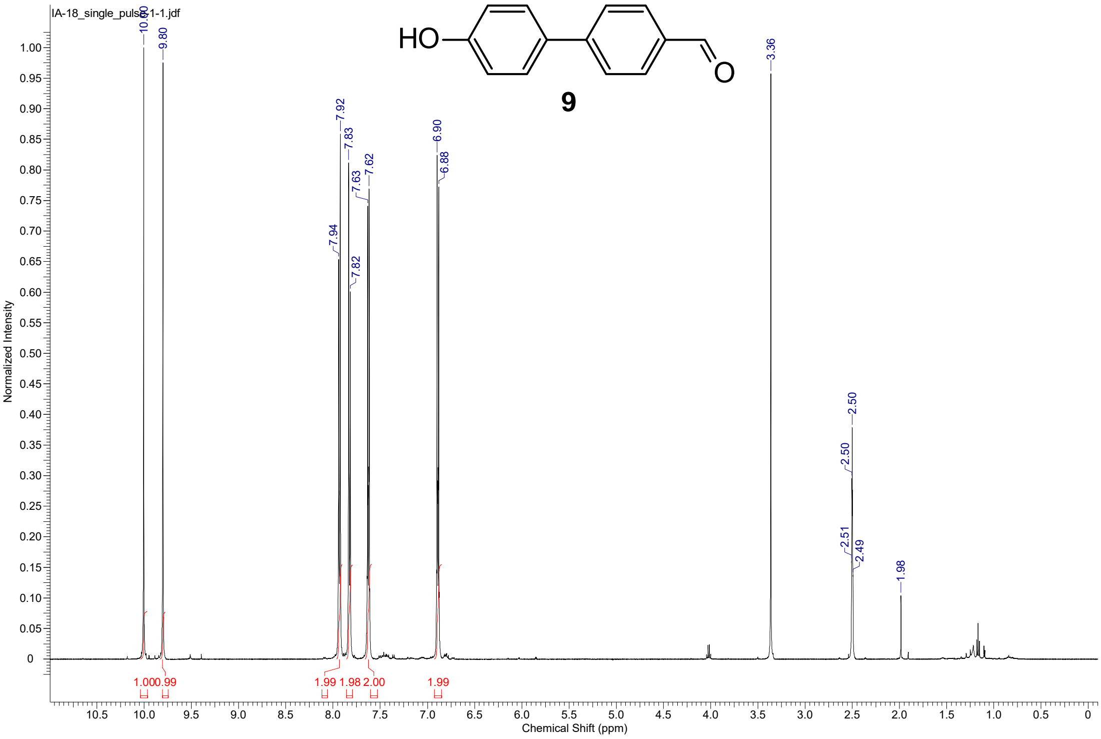

Figure S11. ^{1}H NMR spectra of compound 9.

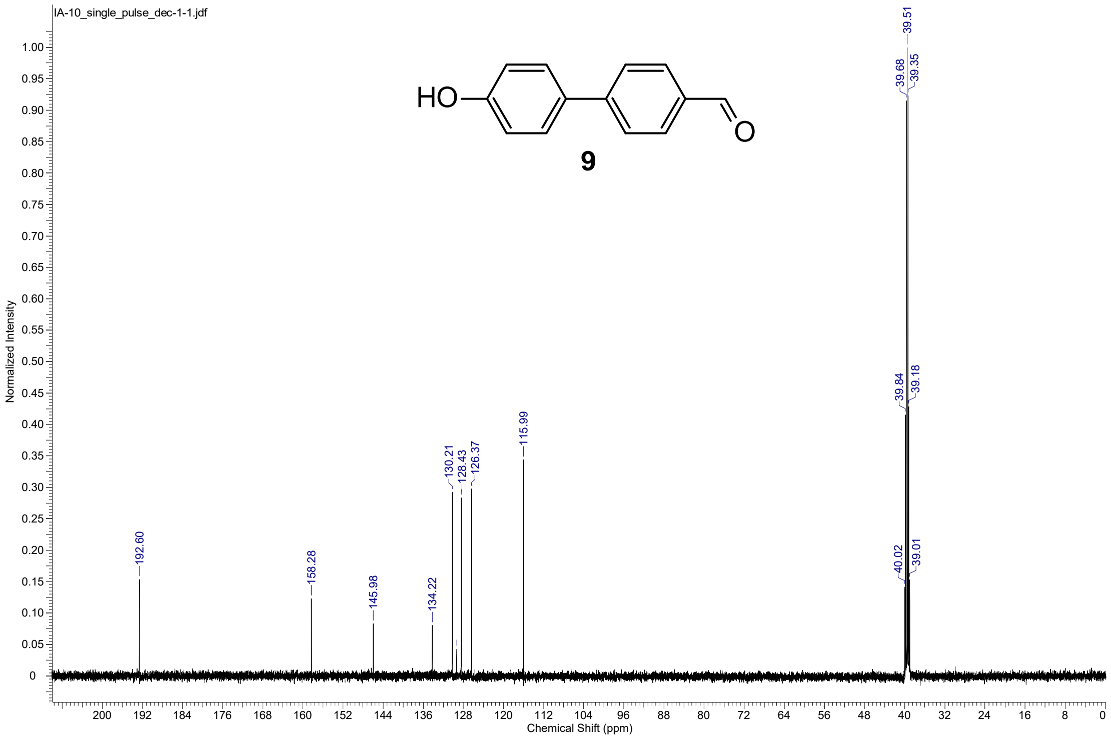

Figure S12. ^{13}C NMR spectra of compound 9.

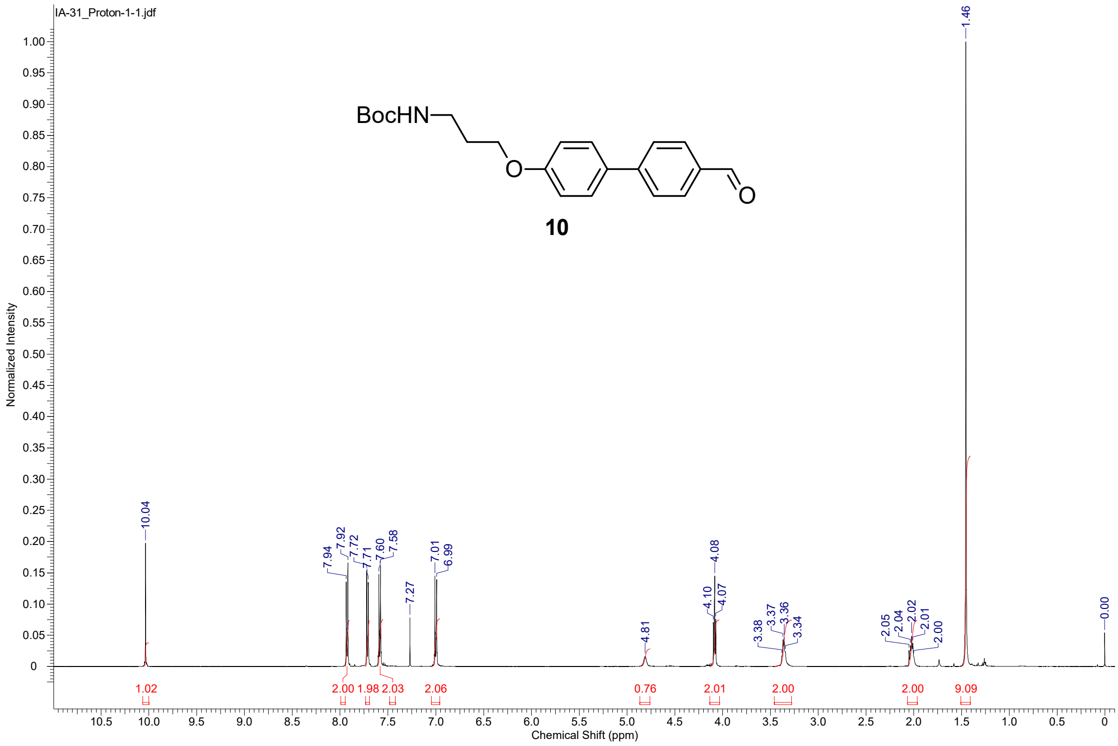

Figure S13. ^{1}H NMR spectra of compound 10.

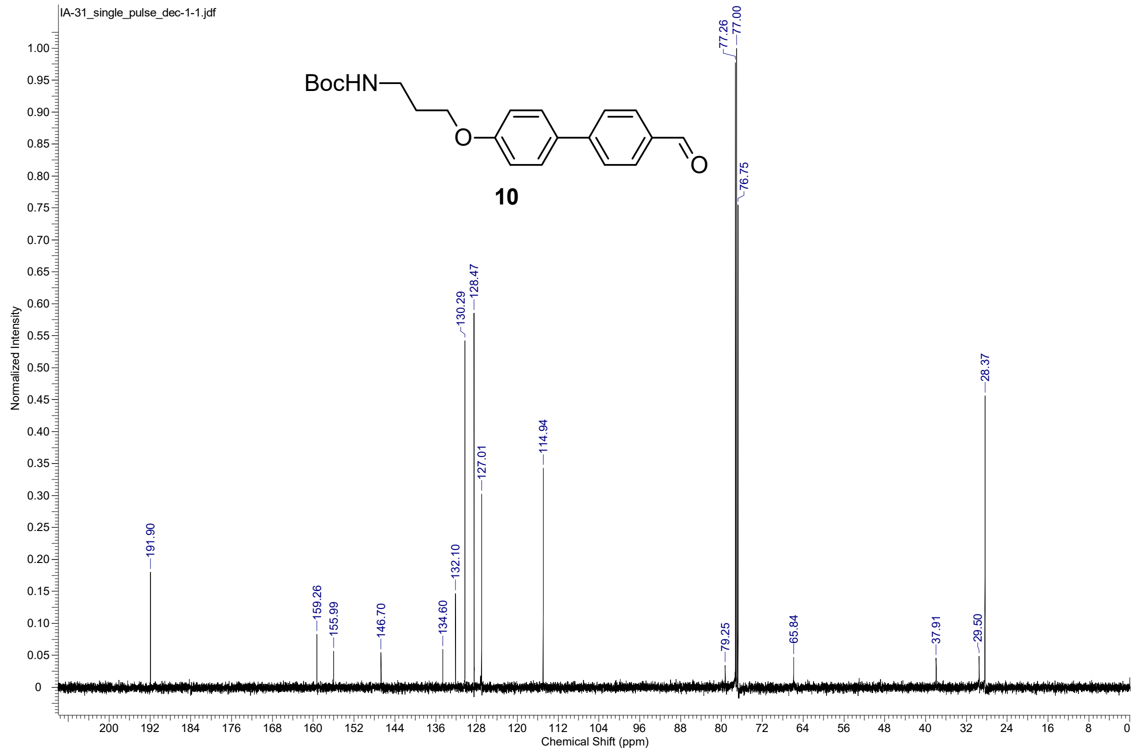

Figure S14. ^{13}C NMR spectra of compound 10.

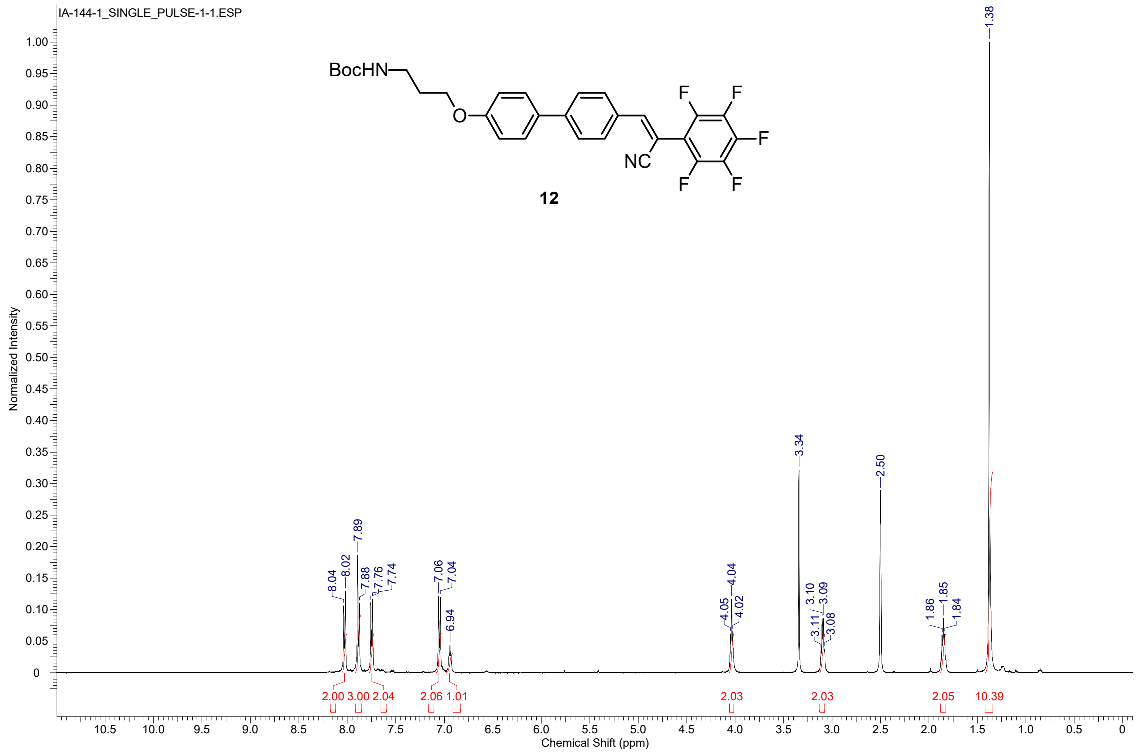

Figure S15. ^{1}H NMR spectra of compound 12.

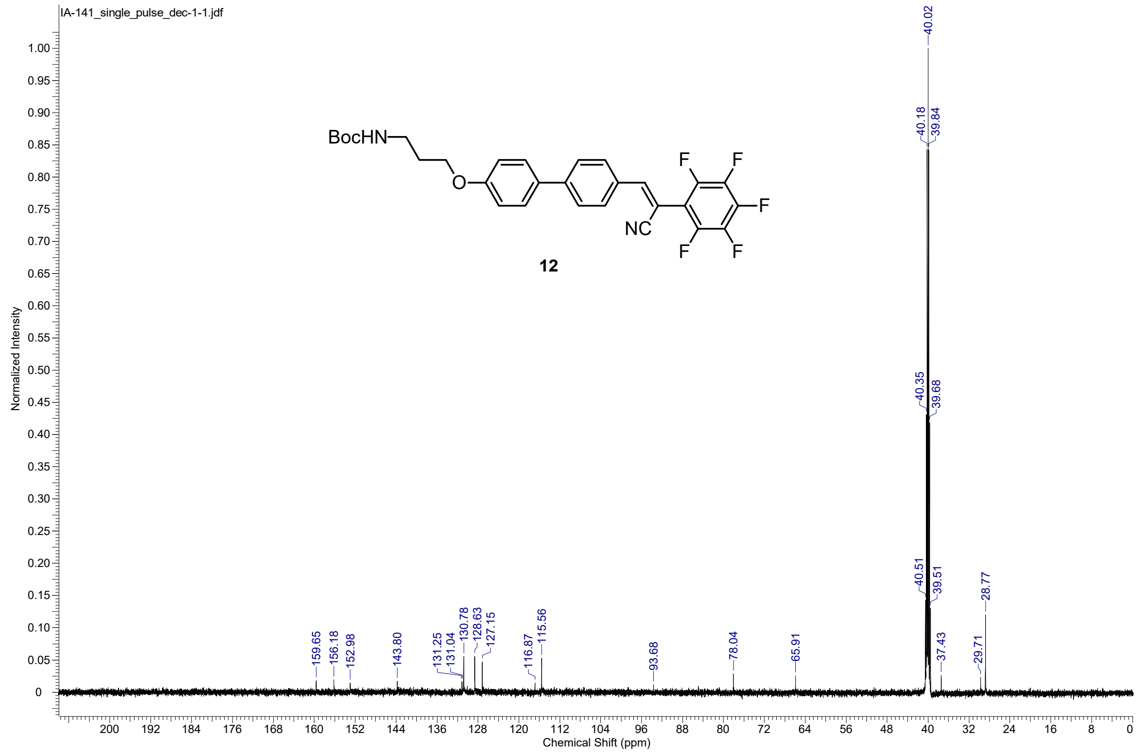

Figure S16. ^{13}C NMR spectra of compound 12.

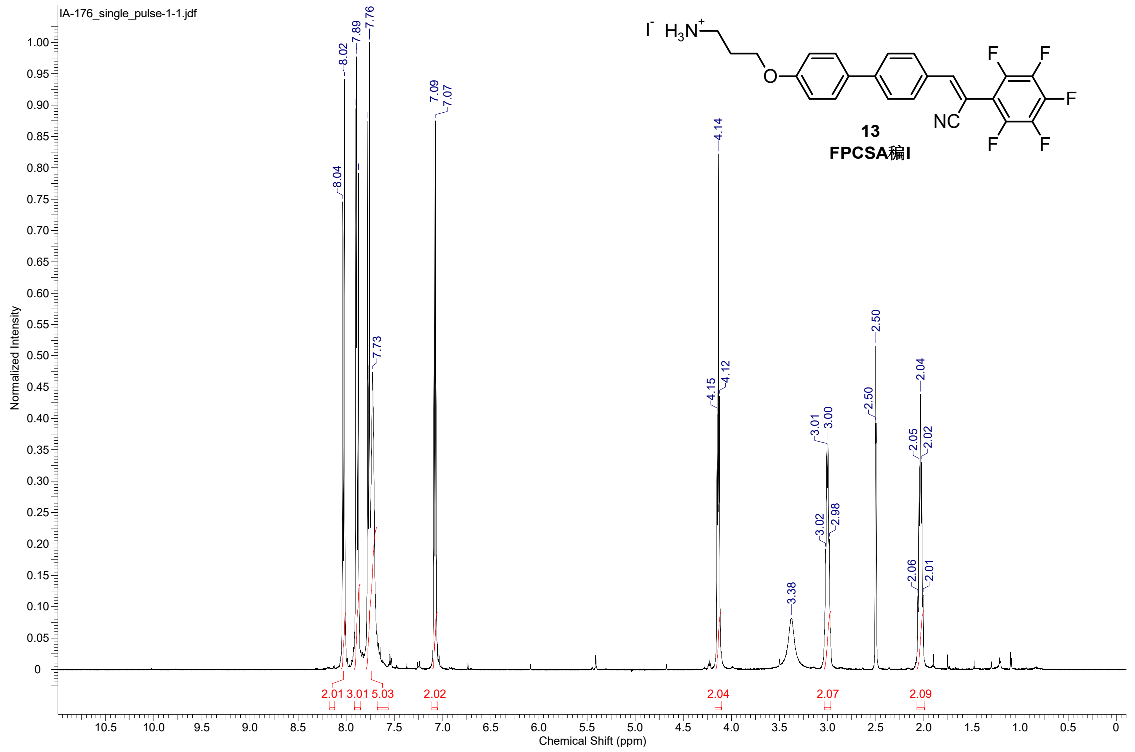

Figure S17. ^{1}H NMR spectra of compound 13.

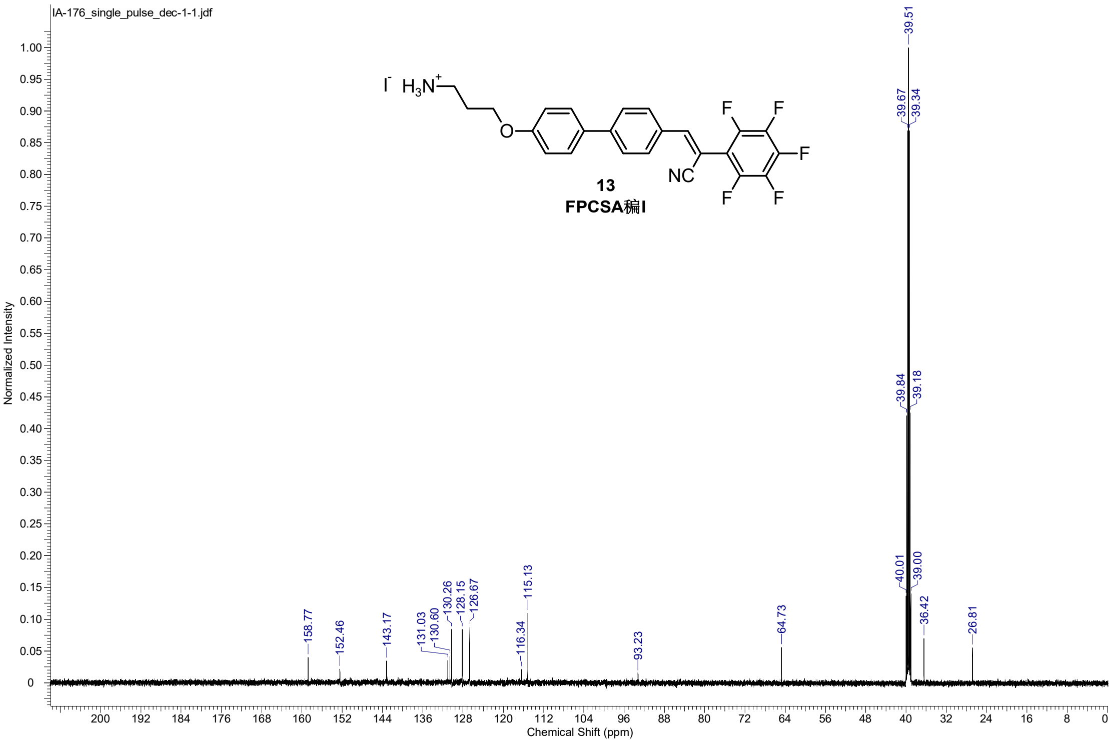

Figure S18. ^{13}C NMR spectra of compound 13.

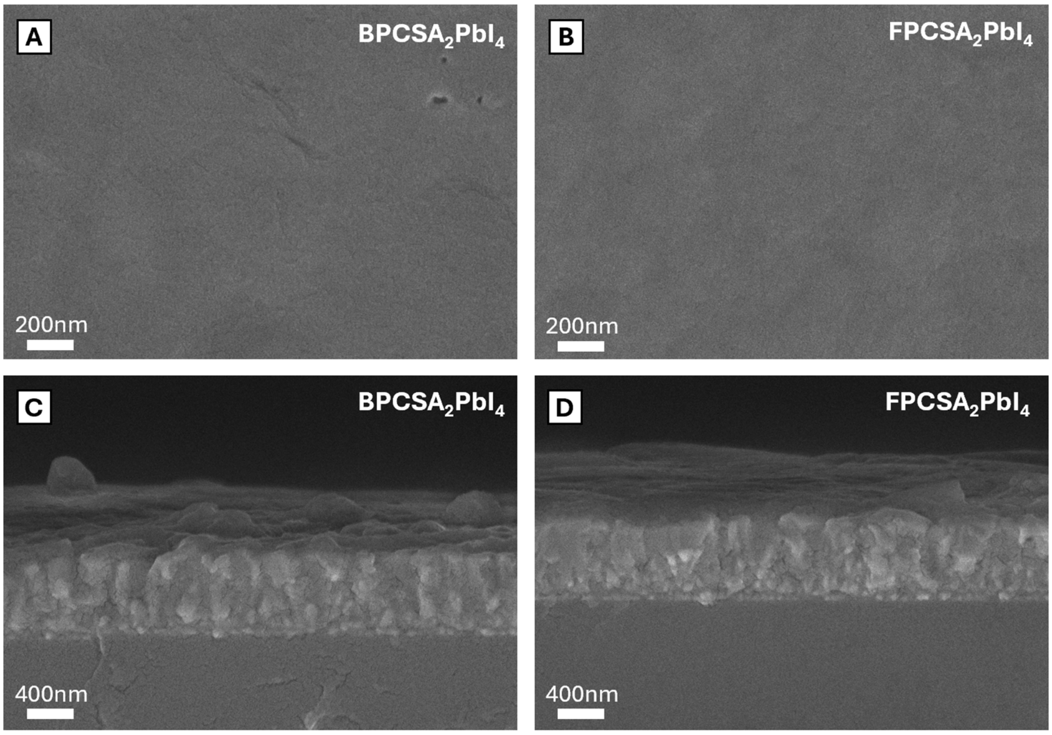

Figure S19. A,B top view and C,D cross-sectional view SEM pictures of $BPCSA_2PbI_4$, and $FPCSA_2PbI_4$ films, respectively.

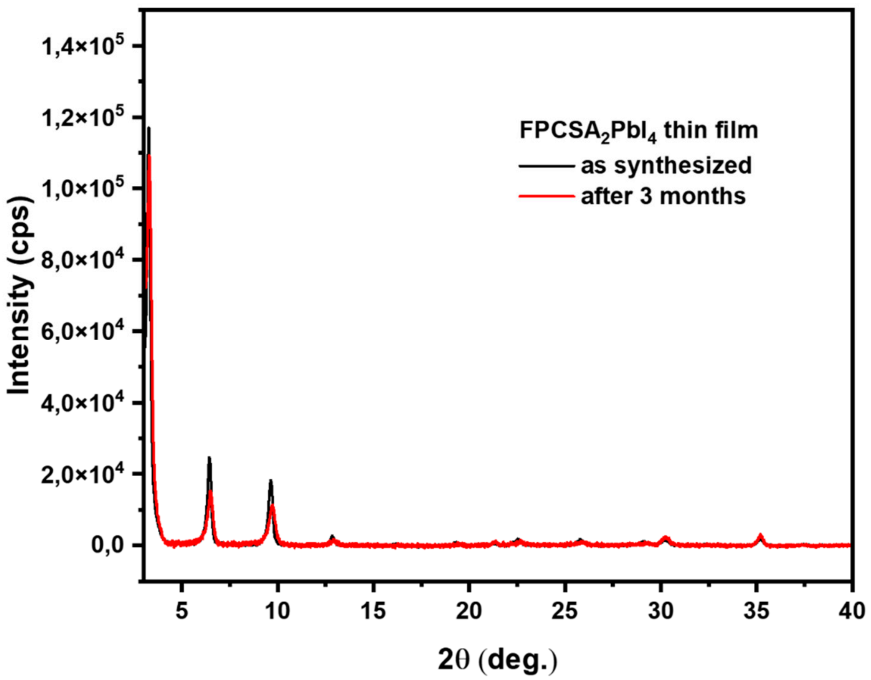

Figure S20. XRD patterns of the as-synthesized FPCSA$_2$PbI$_4$ film and the same film after 3 months.

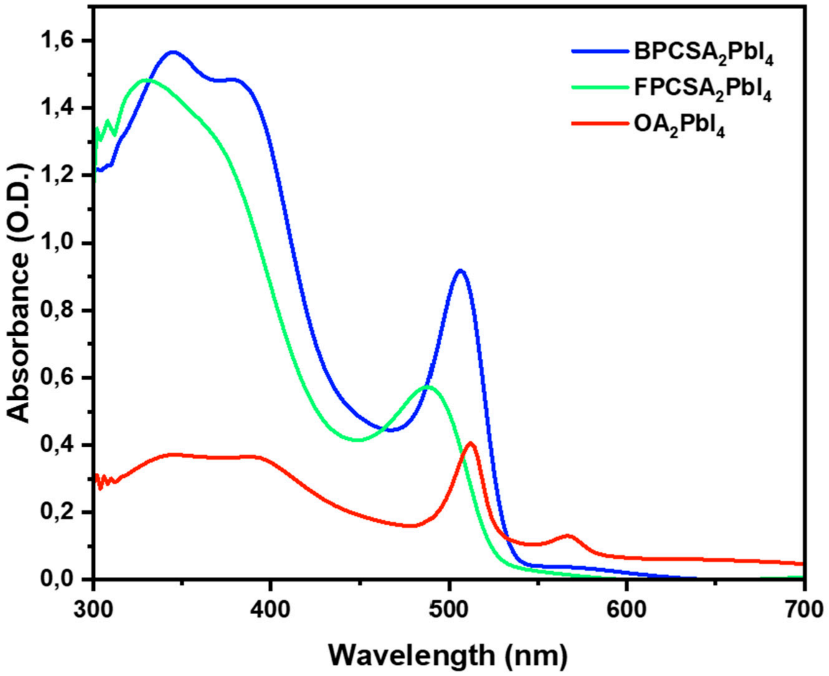

Figure S21. UV-vis spectras of BPCSA$_2$PbI$_4$, FPCSA$_2$PbI$_4$ and OA$_2$PbI$_4$ thin films

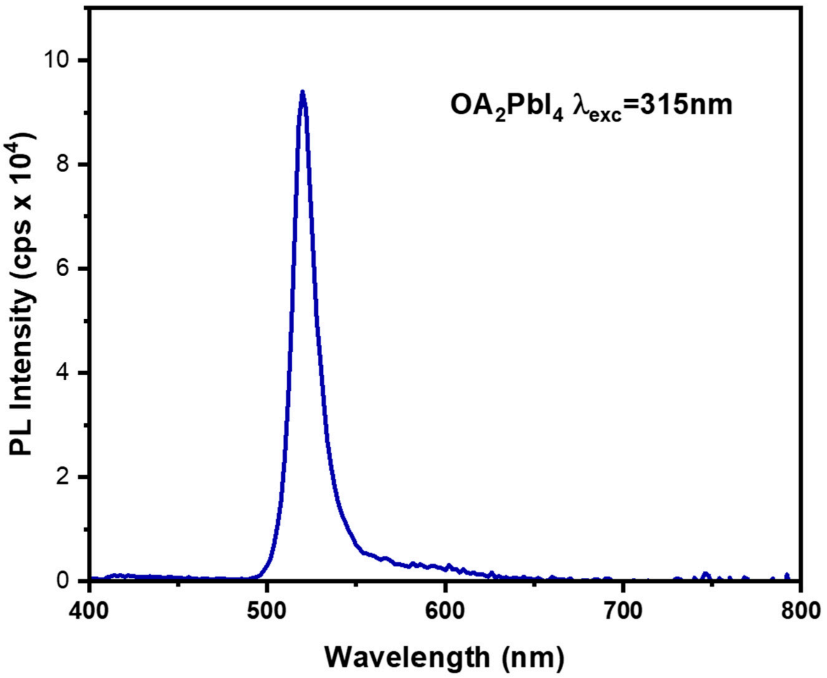

Figure S22. PL emission spectra of OA_2PbI_4 at 315nm excitation
